# High Throughput Determination of TGFβ1/SMAD3 Targets in A549 Lung Epithelial Cells

**DOI:** 10.1371/journal.pone.0020319

**Published:** 2011-05-20

**Authors:** Yingze Zhang, Daniel Handley, Tommy Kaplan, Haiying Yu, Abha S. Bais, Thomas Richards, Kusum V. Pandit, Qilu Zeng, Panayiotis V. Benos, Nir Friedman, Oliver Eickelberg, Naftali Kaminski

**Affiliations:** 1 Division of Pulmonary, Allergy and Critical Care Medicine, Richard P. and Dorothy P. Simmons Center for Interstitial Lung Disease, University of Pittsburgh School of Medicine, Pittsburgh, Pennsylvania, United States of America; 2 Department of Molecular and Cell Biology, California Institute for Quantitative Biosciences, University of California, Berkeley, California, United States of America; 3 School of Computer Science and Engineering, The Hebrew University of Jerusalem, Jerusalem, Israel; 4 Comprehensive Pneumology Center, University Hospital of Ludwig Maximilians University Munich, Asklepios Hospital Gauting, and Helmholtz Zentrum München, Munich, Germany; 5 Department of Computational and Systems Biology, University of Pittsburgh, Pittsburgh, Pennsylvania, United States of America; 6 School of Computer Science and Engineering, Alexander Silberman Institute of Life Sciences, The Hebrew University of Jerusalem, Jerusalem, Israel; University of Sheffield, United Kingdom

## Abstract

**Background:**

Transforming growth factor beta 1 (TGFβ1) plays a major role in many lung diseases including lung cancer, pulmonary hypertension, and pulmonary fibrosis. TGFβ1 activates a signal transduction cascade that results in the transcriptional regulation of genes in the nucleus, primarily through the DNA-binding transcription factor SMAD3. The objective of this study is to identify genome-wide scale map of SMAD3 binding targets and the molecular pathways and networks affected by the TGFβ1/SMAD3 signaling in lung epithelial cells.

**Methodology:**

We combined chromatin immunoprecipitation with human promoter region microarrays (ChIP-on-chip) along with gene expression microarrays to study global transcriptional regulation of the TGFβ1/SMAD3 pathway in human A549 alveolar epithelial cells. The molecular pathways and networks associated with TGFβ1/SMAD3 signaling were identified using computational approaches. Validation of selected target gene expression and direct binding of SMAD3 to promoters were performed by quantitative real time RT-PCR and electrophoretic mobility shift assay on A549 and human primary lung epithelial cells.

**Results and Conclusions:**

Known TGFβ1 target genes such as *SERPINE1, SMAD6, SMAD7, TGFB1* and *LTBP3*, were found in both ChIP-on-chip and gene expression analyses as well as some previously unrecognized targets such as *FOXA2*. SMAD3 binding of *FOXA2* promoter and changed expression were confirmed. Computational approaches combining ChIP-on-chip and gene expression microarray revealed multiple target molecular pathways affected by the TGFβ1/SMAD3 signaling. Identification of global targets and molecular pathways and networks associated with TGFβ1/SMAD3 signaling allow for a better understanding of the mechanisms that determine epithelial cell phenotypes in fibrogenesis and carcinogenesis as does the discovery of the direct effect of TGFβ1 on FOXA2.

## Introduction

Transforming Growth Factor β1 (TGFβ1) is a key pro-fibrotic cytokine involved in many cell signaling and cellular processes. These include cell proliferation, differentiation, cell adhesion and migration, extracellular matrix deposition, apoptosis, embryonic development, and immune response [1], [2], [3], [4], [5], [6], [7]. Dysregulated or aberrant TGFβ1 signaling is implicated in numerous pathological conditions including cancer, pulmonary hypertension, and a wide variety of organ-specific fibrotic diseases, including renal and idiopathic pulmonary fibrosis (IPF) [7], [8], [9], [10].

TGFβ family of proteins is also highly conserved across mammalian species [4], [11]. Ubiquitous expression of both TGFβ and its receptors are detected in nearly all cell types, although the effects on each type of cell are varied and specific to a particular cell type [1], [3], [12], [13], [14]. TGFβ1 exerts its effects through the TGFβ1/SMAD3 signal transduction pathway operating between cell surface receptors for TGFβ1 and the gene regulatory machinery in the nucleus [15], [16]. In humans, there are eight members of the SMAD family of transcription factors. Of these, five are receptor-regulated SMADs, or R-SMADs: SMAD1, SMAD2, SMAD3, SMAD5 and SMAD9. SMAD4 is referred to as a common-mediator SMAD, or co-SMAD. SMAD6 and SMAD7 are antagonistic or inhibitory SMADs and are therefore referred to as I-SMADs [12].

In most cell types, TGFβ1 inhibits cell proliferation [17]. TGFβ1 stimulation of epithelial cells, however, either (a) inhibits cell proliferation, (b) causes cells to undergo apoptosis, or (c) induces *epithelial-mesenchymal transition* or EMT [18], [19], [20], [21]. The mesenchymal cells that result from EMT closely resemble fibroblasts in morphology and behavior, sometimes with additional motile and contractile abilities characteristic of muscle cells (and hence referred to as myofibroblasts) [17], [22]. Prolonged TGFβ1 stimulation induces these mesenchymal cells to secrete collagens such as Collagen 7A1 (COL7A1), decrease protease production, and increase the secretion of protease inhibitors such as TIMPs and SERPINE1, also known as plasminogen activator inhibitor 1 (PAI-1) [4], [5], [23], [24]. Eventually, the cells may begin expressing alpha-smooth muscle actin (αSMA) and transition into motile myofibroblasts that aggressively infiltrate and deposit ECM proteins, particularly collagens [6], [9], [18], [19], [20], [22], [23], [25], [26], [27]. While EMT is expected to occur during certain phases of normal embryonic development, in adults it is characteristic of fibrotic diseases as well as neoplastic invasions and metastasis [9], [22], [27]. The TGFβ1/SMAD3 signal transduction pathway is directly implicated in inducing EMT [10], [28].

Although many genes are known to be regulated through TGFβ signaling pathway, a comprehensive list of genes directly targeted by SMAD3 binding is unavailable. In this study, using a combination of genome-wide technology and computational approaches, we identified SMAD3 target genes and molecular pathways in a human lung alveolar epithelial carcinoma cell line. A novel TGFβ1/SMAD3 target gene, Forkhead Box A2 (*FOXA2*, also known as *HNF3B*), was identified as a direct SMAD3 target. Direct binding of SMAD3 to *FOXA2* was demonstrated in this study. Genome-wide identification of targets and molecular pathways associated with TGFβ1/SMAD3 pathway will provide insights to its function and lead to better understanding of its important roles in multiple cellular processes.

## Materials and Methods

### Cell Cultures

Human lung alveolar epithelial carcinoma A549 cells (CCL-185, ATCC, Manassas, VA) were grown in F12-K culture medium supplemented with 10% fetal bovine serum (ATCC) and subcultured at 80-90% confluency. Prior to all experiments, cells were serum-starved for 18–24 h. Human primary Small Airway Epithelial Cells (SAEC) were obtained from Lonza, Inc. and cultured in serum-free Small Airway Medium with supplied supplements (Lonza, Walkersville, MD).

### Chromatin Immunoprecipitation

The ChIP procedure was performed according to the protocol of Weinmann et al [29] with the following modifications: 1×10^7^ A549 cells were treated with TGFβ1 (2 ng/ml) for up to 2 h. Cells were cross-linked with 1% formaldehyde for 12 min at RT, after which glycine (125 mM) was added to quench the formaldehyde. The cells were washed twice with ice-cold PBS and lysed in 500 µl cell lysis buffer [50 mM Tris-HCl, pH 8.0; 1% Triton X-100; 10 mM KCl; supplemented with complete protease inhibitor cocktail (Roche Diagnostics, Basel, Switzerland)]. Nuclei were pelleted at 2,800×.g for 5 min at 4°C, and resuspended in 400 µl of nuclear lysis buffer (50 mM Tris-HCl, pH 8.0; 10 mM EDTA; 0.1% SDS; supplemented with complete protease inhibitor cocktail). The samples were sonicated 3×10 s to yield sheared DNA fragments between 200 and 700 bp, and lysates were clarified by centrifugation (18,000×. g. 10 min, 4°C). Samples were then incubated with 25 µg of anti-Smad3 antibody or control IgG (anti-flag, Upstate/Millipore, Billerica, MA) for 1 h at 4°C. To reduce nonspecific association, 30 µg of sonicated salmon sperm DNA and 50 µg of BSA (Promega, Madison, WI) were added to each sample. Immunoprecipitation (IP) was carried out using 50 µl of 50% (v/v) Protein A/G PLUS-Agarose beads (Santa Cruz, Santa Cruz, CA) at 4°C overnight. The immune complexes were washed as follows: three times with low-salt wash buffer (10 mM Tris-HCl, pH 8.0; 0.1% SDS; 0.1% sodium deoxycholate; 1% Triton X-100; 1 mM EDTA; 140 mM NaCl), 3 times with high-salt buffer (same as low-salt wash buffer, but with 500 mM NaCl), 2 times with LiCl wash buffer (10 mM Tris-HCl, pH 8.0; 250 mM LiCl; 1% Nonidet P-40; 1% sodium deoxycholate; 1 mM EDTA), and 2 times with TE buffer (20 mM Tris-HCl, pH 8.0; 1 mM EDTA). Elution was performed twice at 65°C for 15 min, first with 200 µl of 1.5% SDS solution, and then with 250 µl of 0.5% SDS solution. Immunoprecipitated DNA-protein complexes were then reverse cross-linked at 65°C overnight and purified by phenol-chloroform extraction and ethanol precipitation with 30 µg glycogen (Roche Diagnostics). The resultant purified DNA was dissolved in 20 µl of water.

### Promoter Microarrays

Purified nucleic acid of ChIP reactions was blunt-ended using T4 DNA polymerase and ligated to linkers (sense strand: 5′-GCGGTGACCCGGGAGATCTGAATTC-3′; anti-sense strand: 5′-GAATTCAGATC-3′) using T4 DNA ligase. Ligation products were amplified using a two-stage (15 cycles followed by dilution and input to a 25 cycle reaction) *Taq* polymerase-based PCR and purified using PCR reaction purification kit (Qiagen, Valencia, CA). Purified PCR products of SMAD3 IP and Mock IP were labeled with cyanine-5 (Cy5) and cyanine-3 (Cy3) florescent dyes (PerkinElmer, Boston, MA), respectively, using the BioPrime® Array CGH Genomic Labeling kit (Invitrogen, Carlsbad, CA). Dye incorporation was verified by Nanodrop spectrophotometer measurement (Nanodrop, Wilmington, DE). Labeled amplified DNA (Cy5 and Cy3) was combined and hybridized to Agilent 44K two-array whole genome promoter sets (Agilent, Santa Clara, CA) for 40 h at 65°C. Arrays were then washed in a series of sodium chloride-sodium citrate (SSC) buffers and acetonitrile, and treated with Agilent stabilization and drying solution for 30 seconds. Arrays were then immediately scanned on a GenePix 4000B scanner in two-color array mode (Cy5/Cy3) yielding an intensity ratio of Cy5 (IP) to Cy3 (mock IP) for each probe.

### ChIP-on-chip Promoter Microarray Analysis

Agilent 44K whole genome promoter arrays contain probes that cover 2000 base pair upstream to 800 base pair downstream of the transcriptional start site for 44,000 published RefSeq genes. The probed areas contain on average four to six separate 60-mer sequences spaced at approximately 300 base pair intervals. Human genome assembly UCSC hg17 which was built based on human genome assembly NCBI35 was used for all genomic analyses (http://genome.ucsc.edu/).

For Agilent promoter microarray analysis, we used a model-based algorithm developed by Capaldi *et. al* [30]. Briefly, the algorithm uses the length distribution of DNA fragments (after sonication) to estimate the shape of a single binding event, as measured by a series of 60-mer probes in each promoter sequence on the array. Once the shape of a binding event at the ChIP-on-chip data is modeled, the method then identifies regions of high occupancy and optimizes the peak position and height (relative enrichment) by fitting the peak shape to the measured data. For each peak, the algorithm enumerates and selects the most probable values for center position and peak height (enrichment) and computes the statistical significance of this peak. The statistical significance of a binding event is estimating by computing an empirical log-likelihood ratio (LLR) *p*-value. In this study, a binding event was defined by a *p*-value less than 0.01 and an estimated peak height (enrichment over control background) greater than 1.5. The peak height was a measurement of enrichment of specific SMAD3 binding to target sequences.

We analyzed SMAD3 ChIP-on-chip data for peaks with and without TGFβ1 stimulation. Each peak was assigned an enrichment value and a *p*-value (the statistical significance of seeing such a peak at random). To differentiate the true target genes of SMAD3 with and without TGFβ1 stimulation, we analyzed the ChIP-on-chip data, and identified genes whose promoter was bound by SMAD3 in at least two of the three array replicates. For this, we used a *p*-value threshold of 0.01 in each of the two replicates. For all peak regions, ±100bp surrounding the peak position were extracted for further analysis. Computational scanning for SMAD3 binding sites was performed using existing SMAD3 matrix from TRANSFAC (release 2010.1, matrix identifier V$SMAD3_Q6) and UniPROBE [31], [32] (UniPROBE Accession number UP00000) as shown in Figure S1. Bound regions were scanned for matrix hits for each of the matrices using a type I cutoff calculated at a p-value of 0.05 [33] where the background score distribution was approximated by sampling 10000 instances of the motif length from the uniform distribution and scoring using the motif matrix. The ChIP-on-chip microarray data was deposited to the GEO database under the accession number GSE28346 (http://www.ncbi.nlm.nih.gov/geo/).

### Gene-Specific PCR Verification

A portion of the ligation-mediated PCR amplified immunoprecipitation product was used for gene-specific PCR reactions (25 cycles) to verify enrichment of promoter regions of the known TGFβ1-responsive genes *SERPINE1* and *SMAD7* as well as the *FOXA2* promoter sequence. PCR was performed using *Taq* DNA polymerase (Invitrogen) in 15 µl reactions according to the manufacturer's protocol.

### Gene Expression Microarrays

For gene expression measurements we used Agilent 4×44K whole human genome microarray kits (Agilent, Santa Clara, CA) according to the manufacturer's instructions. Briefly, 500 ng of total RNA was amplified using an Agilent Low Input Linear Amplification and Labeling kit and resultant cRNA was labeled with Cy3 (10 mM; PerkinElmer, Boston, MA). Cy-3 labeled probes were purified using Qiagen RNeasy Mini kit (Qiagen) according to the manufacturer's protocol. The yield and dye incorporation were confirmed using a Nanodrop spectrophotometer (Nanodrop, Wilmington, DE). Arrays were hybridized for 17 h at 60°C under continuous rotation at ∼20 RPM. The gasket slide coverslips were removed and the slides were sequentially washed with Agilent Wash Buffer 1, Agilent Wash Buffer 2, and acetonitrile for 1 min each and stabilized for 30 seconds in Agilent Stabilization and Drying solution. Arrays were scanned using the Agilent DNA microarray scanner.

DNA microarray feature intensities were measured using Agilent Feature Extraction software 9.5.2. There were three replicates each of four time points (0, 2, 12, and 24 h) of TGFβ1 stimulation, each for vehicle-only control (DMSO) and for SIS3 treatment.

### SIS3 Inhibition of SMAD3 Activity

Specific Inhibitor of SMAD3 (SIS3, EMD Chemicals, Inc., San Diego, CA) is a potent, specific inhibitor of TGFβ1/ALK-5 phosphorylation of SMAD3 while having no effect on SMAD2, p38 MAPK, ERK, or PI 3-K signaling [34]. Cultured A549 cells at 30-50% confluence were treated with 10 uM SIS3 in dimethyl sulfoxide (DMSO), or DMSO (vehicle-only) 30 min prior to TGFβ1 treatment. Cells were treated with 2 ng/mL recombinant TGFβ1 (R&D Systems, Minneapolis, MN) for 0, 2, 12, and 24 h. Total mRNA was extracted using Trizol (Invitrogen) according to the supplier's protocol.

### Expression Microarray Data Analysis and Statistics

Background-subtracted signal intensities of arrays were log-base 2 transformed and then normalized across arrays by cyclic loess in the R statistical package (R-2.6.0). Briefly, cyclic loess normalization as used here involves randomly selecting a subset of 5000 probes for the cyclical fitting of local linear smoothers (loess from the stats package) to MA plots from pairs of arrays, with response variable M (log_2_-transformed intensity ratio) and independent variable A (log_2_ of geometric mean intensity), then adjusting the intensity values of all probes on both arrays in the pair using predicted values from the loess fit. Each iteration is a complete cycle over all pairs of arrays in the data set, and iteration stops once the maximum observed change is less than a specified epsilon, usually only two or three iterations. Since array data often contains multiple (and variable numbers of) probes per gene, the probe intensities were averaged and combined into individual gene intensity values. Individual gene intensities across arrays (*i.e.,* row) were geometric mean normalized to the first time point (0 h control) [35], [36], [37], [38], [39], [40].

The data were analyzed using three separate software packages: first, by permutation test between separate time points in the R statistical programming environment (www.r-project.org/); next by the Significance Analysis of Microarrays (SAM) package from Stanford (www-stat.stanford.edu/~tibs/SAM/) [41]; and finally in the Short Time-series Expression Miner (STEM) package [42], [43]. The STEM program uses a permutation test to quantify the expected number of genes that would have been assigned to each model if the data were random. Thus, a gene expression profile deemed as significant would generate an established pattern similar to other genes in its group and distinctly different from random deviation. The resultant *p*-values are then Bonferroni corrected [42], [43]. The gene expression microarray data was deposited to the GEO database under the accession number GSE26858 (http://www.ncbi.nlm.nih.gov/geo/).

### Electrophoretic Mobility Shift Assay (EMSA)

Cultured A549 at 60–70% confluence were treated with 2 ng/ml recombinant human TGFβ1 (R&D Systems, Minneapolis, MN) for 60 min. Nuclear proteins were isolated as described previously [44]. Nuclear proteins were flash-frozen in liquid nitrogen and stored at −80°C.

Nuclear extracts at 1∶10 dilution and recombinant full length SMAD3 protein (Santa Cruz, Santa Cruz, CA) were incubated with 5′-end Cy5 labeled probe and/or non-labeled competitor oligonucleotide for 20 min at room temperature in a binding buffer consisting of 20% glycerol, 5 mM MgCl2, 2.5 mM EDTA, 25 mM DTT, 200 mM NaCl, 50 mM Tris HCl pH 7.6, and 0.25 mg/mL poly(dI-dC). The oligonucleotides (5′-Cy5-GATTGCTGGTCGTTTGTTGTGGCT-3′, 5′-AGCCACAACAAACGACCAGCAATC- 3′) consisted of nucleotide -42 to -19 relative to the translation start site of *FOXA2* promoter were synthesized (IDT, Coralville, IA). Supershift assay was performed by additionally incubating nuclear extract with 0.4 µg rabbit polyclonal antibody to SMAD3 (Abcam, Cambridge, MA) prior to incubating with oligonucleotide. The protein/DNA complexes were run on a 6% native polyacrylamide gel and visualized on a Typhoon 9400 imaging and documentation system using Cy5dye excitation and fluorescence settings.

### Quantitative Real-Time PCR

A549 cells and human Small Airway Epithelial Cells were grown to 80–90% confluence and treated with 2 ng/mL recombinant TGFβ1 for 0 (control), 2, 12, and 24 h. Total mRNA was extracted using Trizol (Invitrogen) according to the manufacturer's instruction. Total mRNA was normalized to 600 ng and reverse-transcribed using random hexamer priming with a SuperScript kit (Invitrogen). Quantitative PCR was performed using TaqMan Gene Expression Assays specific for *FOXA2* (Hs00232764_m1) and *SERPINE1* (Hs01126604_m1) on an ABI Prism 7900HT (Applied Biosystems, Foster City, CA). To evaluate relative mRNA expression of *FOXA2* and *SERPINE1*, we used *GAPDH* as a reference gene. Relative changes in transcript levels of *FOXA2* and *SERPINE1* as compared to controls are expressed as ΔΔCt values (ΔΔCt  =  ΔCttreated – ΔCtcontrol) using ABI Sequence Detection Software v2.2.2.

### Functional Analysis

#### Network Generation

A data set of significantly bound (ChIP) or up/down-regulated (expression) genes containing gene identifiers and corresponding binding/expression values was uploaded into Ingenuity. Each gene identifier was mapped to its corresponding gene object in the Ingenuity Pathways Knowledge Base [45]. These genes, called focus genes, were overlaid onto a global molecular network developed from information contained in the Ingenuity Pathways Knowledge Base. Networks of these focus genes were then algorithmically generated by Ingenuity Pathways Analysis based on their connectivity.

#### Functional Analysis of a Network

The Functional Analysis of a network identified the biological functions that were most significant to the genes in the network. The network genes associated with biological functions and/or diseases in the Ingenuity Pathways Knowledge Base were considered for the analysis. Fisher's exact test was used to calculate a *p*-value determining the probability that each biological function assigned to that network is due to chance alone.

#### Canonical Pathway Analysis

Canonical pathways analysis identified the pathways from the Ingenuity Pathways Analysis library of canonical pathways that were most significant to the data set. A data set of significantly bound (ChIP) or up/down-regulated (expression) genes containing gene identifiers and corresponding binding/expression values was uploaded into in the application and associated with a canonical pathway in the Ingenuity Pathways Knowledge Base. The significance of the association between the data set and the canonical pathway was measured in two ways: 1) A ratio of the number of genes from the data set that map to the pathway divided by the total number of genes that map to the canonical pathway is displayed. 2) Fisher's exact test was used to calculate a *p*-value determining the probability that the association between the genes in the dataset and the canonical pathway is explained by chance alone. Analyses were also done using MetaCore GeneGo systems biology tools and default parameters of the software. The detailed methods are described at GeneGo (http://www.genego.com/metacore.php) and elsewhere [46], [47].

## Results

### Identification of SMAD3 Target Genes by ChIP-on-chip

**Figure 1:**
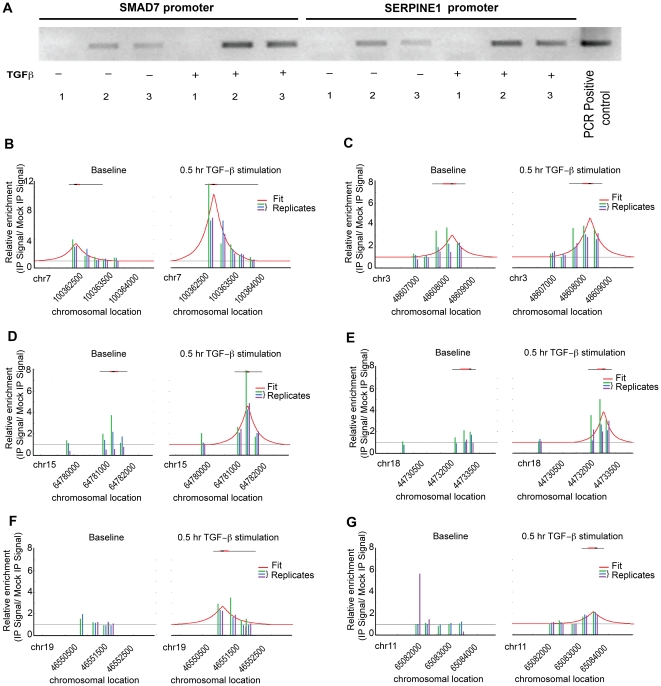
Enhanced SMAD3 binding to known target genes through TGFβ1 stimulation. A: *SMAD7* and *SERPINE1* promoters were amplified using the ligation-mediated PCR amplified immunoprecipitation product and gene specific primers. (1) Mock IP (anti-flag Ab); (2) anti-SMAD3 Ab (Upstate Biosciences); (3) anti-SMAD2,3 Ab (BD Biosciences). B-G: Enhanced SMAD3 binding to target promoters through exogenous TGFβ1 stimulation. The left panel illustrates baseline promoter binding of SMAD3 and the right panel shows promoter binding after 30 min 2 ng/mL TGFβ1 stimulation. The known SMAD3 target genes *SERPINE1*, *COL7A1*, *SMAD6*, *SMAD7*, *TGFB1*, and *LTBP3* are shown in B-G, respectively.

To ensure the success of SMAD3-specific ChIP-on-chip analysis, we first confirmed the sensitivity and specificity of SMAD3-specific ChIP assay. Gene-specific amplifications of two well known direct targets of *SMAD3*, *SMAD7* and *SERPINE1* were performed using PCR and the SMAD3-specific ChIP products of human A549 cells (Figure 1A). *SMAD7* and *SERPINE1* were detected in the products of two independent ChIP assays with different antibodies specific for SMAD3. As expected, TGFβ1 enhanced SMAD3 binding to both promoters. For the ChIP-on-chip analysis, binding peaks were identified by the model-based method of Capaldi *et. al* [30] and significant binding was defined as any peak height of at least 1.5. A total of 350 and 469 genes met the binding criteria at the basal level and after 30 min TGFβ1 stimulation, respectively (Table S1 and S2). The promoter with most abundant binding of SMAD3 after TGFβ1 stimulation was *SERPINE1* with a relative peak height of 3.47 and 10.40 for basal condition and TGFβ1 stimulation, respectively, Similarly, the binding intensities were increased by TGFβ1 stimulation for additional known TGFβ1-responsive genes including *COL7A1*, a component of extracellular matrix, *SMAD6* and *SMAD7*, inhibitory SMAD proteins involved in inhibiting intracellular effects of TGFβ signaling, TGFβ1, and Latent Transforming Growth Factor Binding Protein 3 (LTBP3), TGFβ1 binding protein (Figure 1b). In addition, enhanced binding of SMAD3 by TGFβ1 to transgelin (*TAGLN*), a previously reported TGFβ1/SMAD3 target and marker of EMT and cell mobility, was also detected [48]. Impressively, scanning for matches to known SMAD3 matrices revealed that 70% of the sequences bound by SMAD3 only after TGFβ1 induction had the Smad3 canonical motif (for the TRANSFAC matrix (Figure S1); 57% for the primary SMAD3 matrix from UniPROBE), while 80% had the previously reported alternative Smad3 GC-rich binding motif [49] (for the secondary SMAD3 matrix from UniPROBE).

### Correlation of Promoter Binding by SMAD3 and Altered Gene Expression by TGFβ1

**Figure 2:**
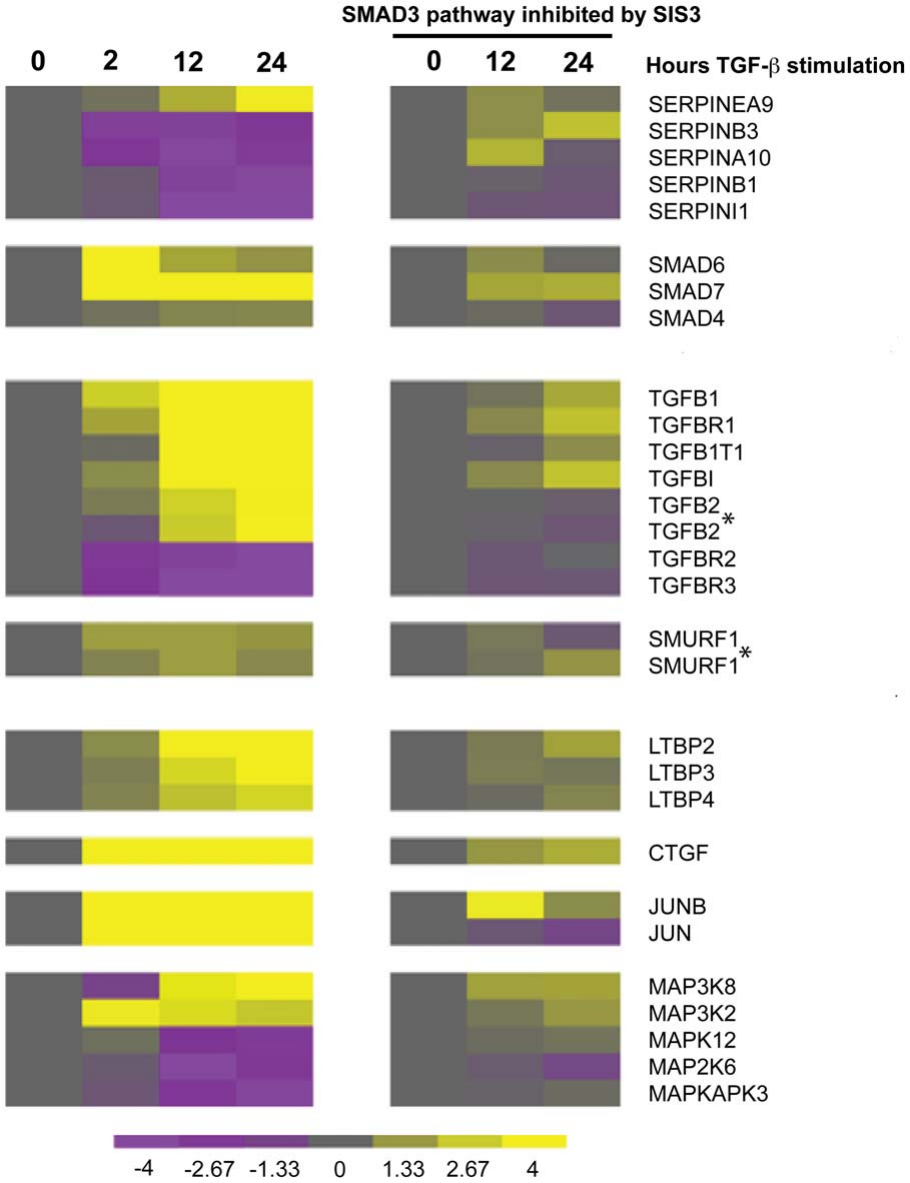
Gene expression levels of known TGFβ1/SMAD3 target genes. Heat map of average expression values for genes known to be affected by the TGFβ1/SMAD3 pathway by microarray analysis. Color intensity values correspond to log_2_ of absolute intensity and reach saturation on the heat map at value 4 to preserve dynamic range at lower values. The time series is in hour after TGFβ1 stimulation and vehicle only (DMSO; left) and with TGFβ1 stimulation and also inhibition of SMAD3/ALK5 phosphorylation by Specific Inhibitor of SMAD3 (SIS3) (right). The gene expression profiles on the left (non-SIS3-treated) were all identified as significantly up- or down-regulated (p<0.00001) by STEM as described in the method section. A * indicated microarray results from two distinct DNA probes of the same gene.

In addition to SMAD3 specific ChIP-on-chip, global gene expression was also analyzed using A549 with or without TGFβ1 stimulation. Gene expression microarray results were consistent with many of the known TGFβ1/SMAD3-responsive elements (Figure 2, left panel). As shown in these heat maps, *SERPINE1*, *SMAD6*, *SMAD7*, *TGFβ1*, SMAD specific E3 ubiquitin-protein ligase 1 (*SMURF1*), a ubiquitin ligase that is specific for receptor-regulated SMAD proteins in the bone morphogenetic protein (BMP) pathway, and Connective Tissue Growth Factor (*CTGF*) were highly upregulated after TGFβ1 simulation. To determine whether these TGFβ1 effects were a direct result of SMAD3 function, we used SIS3, a specific inhibitor of TGFβ1 induced SMAD3 phosphorylation (Figure 2, right panel). Addition of SIS3 reversed the effects of TGFβ1 on these target genes and the degrees of these inhibitory effects were gene specific.

**Figure 3:**
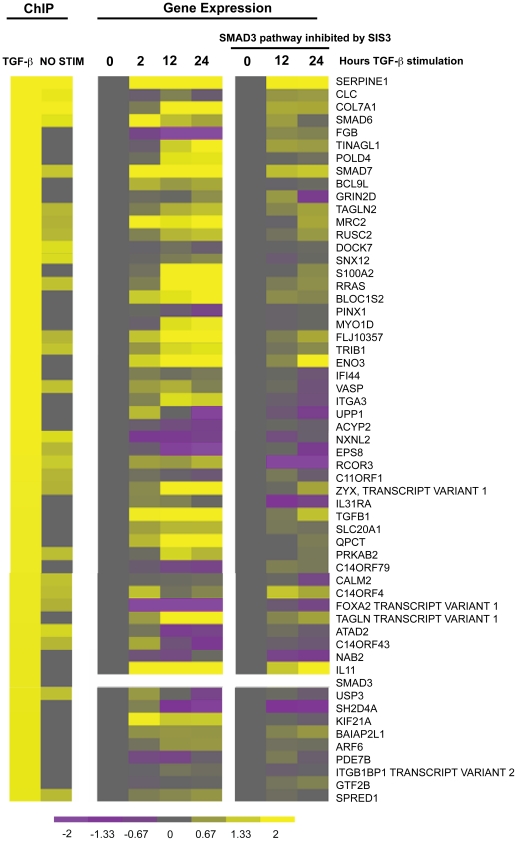
Correlation of promoter binding by SMAD3 and altered gene expression by TGFβ1. Heat map illustrate the genes with highest ChIP binding values (left-most heat map columns) before and after TGFβ1 treatment alongside their respective gene expression microarray intensities (middle heat map columns). Color intensity values correspond to log_2_ of absolute intensity and reach saturation on the heat map at value 4 to preserve dynamic range at lower values. Pre-treatment of A549 cells with SIS3 is shown to attenuate the TGFβ1 gene expression response (right-most heat map columns).

To correlate SMAD3-bound target genes identified by ChIP-on-chip and gene expression analysis, we have analyzed the top 57 genes with the highest changes in the ChIP binding values and their corresponding gene expression at 0, 2, 12, and 24 h TGFβ1 treatment (Figure 3). Generally, the expression levels of the genes with the highest binding index on ChIP-on-chip were changed on the gene expression microarray. Although the expression levels of most target genes were up-regulated by TGFβ1, down regulation of target gene expressions were observed in *FOXA2*, fibrinogen beta chain (*FGB*), epidermal growth factor receptor pathway substrate 8 (*EPS8*), and phosphodiesterase 7B (*PDE7B*). FOXA2 is a known transcription factor in lung development. The repression of *FOXA2* expression was observed at 2 h post treatment and persisted throughout the induction. Addition of SIS3 abolished most of the stimulatory/inhibitory effects of exogenous TGFβ1 administration.

### Signal Pathways of the SMAD3-bound Target Genes

**Figure 4:**
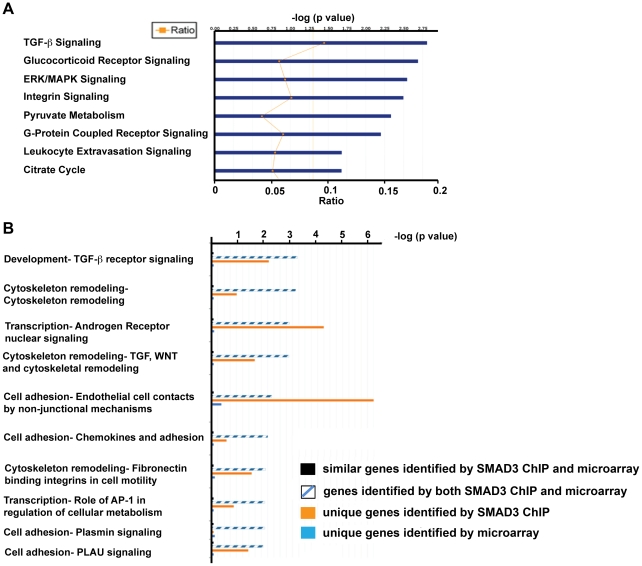
Signal pathways of the SMAD3-bound target genes. A: ChIP SMAD3-bound target genes grouped by signaling pathway and ranked in order of statistical significance. The ratio of genes (orange line) refers to number of genes involved in pathway divided by total genes; approximately 10% of bound genes are identified as belonging to the known TGFβ1 signaling pathway. Other prominent signaling pathways include ERK/MAPK and integrin signaling, which is consistent with known interactions of TGFβ1. Data and image generated using Ingenuity Pathways Analysis. B: Combined gene expression microarray and ChIP-on-chip data grouped by biological process using MetaCore GeneGo systems biology analysis tools [46], [47]. The top 10 identified pathways are shown. The solid blue and orange bars represent the -log *(p*-value) for unique genes identified by the TGFβ1-induced gene expression and the ChIP SMAD3-bound genes, respectively. The stripped blue bars represent the -log (*p*-value) for common genes identified by both TGFβ1-induced gene expression and ChIP SMAD3-bound genes. The black bars represent the -log (*p*-value) for similar genes identified by both TGFβ1-induced gene expression and ChIP SMAD3-bound genes.

We have performed Ingenuity Pathways Analysis to identify signal pathways associated with the SMAD3-bound target genes of TGFβ1 stimulated human A549 cells (Figure 4A). The most important signal pathway was TGFβ signaling and it included approximately 10% of bound genes. Other prominent signaling pathways included glucocorticoid receptor, ERK/MAPK and integrin signaling, which were consistent with known interactions of TGFβ1. In addition, pyruvate metabolism, G-protein coupled receptor signaling, leukocyte extravasation signaling and citrate cycles were also identified. A combined analysis of gene expression microarray and ChIP-on-chip of TGFβ1 treated A549 cells was performed using MetaCore GeneGo system biology analysis tools (Figure 4B). Among the top biological pathways identified by both ChIP-on-chip and microarray, TGFβ receptor signaling remained to be the most significant associated pathway. Interestingly, three of the top 10 pathways were associated with cytoskeleton remodeling (pathways 2, 4, and 7) and 4 of them were associated with cell adhesion related pathways (pathways 5, 6, 9, and 10).

### Validation of Gene Expression Changes by Quantitative Real Time PCR

**Figure 5:**
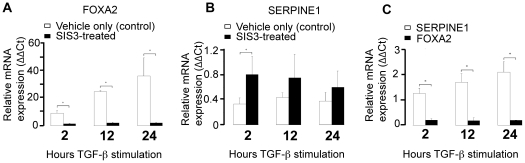
Quantitative analysis of *SERPINE1* and *FOXA2* gene expression. A and B: Quantitative real-time PCR of *SERPINE1* (A) and *FOXA2 (B)* gene expression levels in human A549 cells after 2, 12, and 24 h of stimulation with 2 ng/ml exogenous TGFβ1 and the specific SMAD3 inhibitor, SIS3, or a vehicle-only control (DMSO). The asterisk denotes a highly statistically significant (*p*<0.001; n = 3) difference at each time point between SIS3-treated and vehicle-only controls after TGFβ1 treatment. C: Quantitative real-time PCR of *SERPINE1* and *FOXA2* levels in human Small Airway Epithelial Cells (SAEC) at 2, 12, and 24 h TGFβ1 treatment in relation to control (no TGFβ1). The asterisk denotes a statistically significant (*p*<0.01; n = 3) difference at each time point for *SERPINE1* and for *FOXA2* at 24 h with respect to no TGFβ1 treatment (time zero).

To verify the efficacy of both TGFβ1 stimulation as well as the inhibitory efficiency of SIS3 treatment in A549 cells, mRNA levels of the highest responsive gene *SERPINE1* and *FOXA2*, one of the down regulated genes, were determined by quantitative real-time PCR (Figure 5A and 5B). *SERPINE1* levels increased approximately 10, 25 and 36 folds at 2, 12, and 24 h TGFβ1 stimulation. Conversely, *FOXA2* levels were repressed by approximately 70-80% at 2, 12, and 24 h. The stimulation and repression effects were largely abrogated by SIS3 treatments, suggesting that it was mediated specifically and directly through the TGFβ1/SMAD3 pathway. To assess whether these effects were specific only to the A549 cell line, *SERPINE1* and *FOXA2* mRNA levels were also measured in primary human small airway epithelial cells (SAEC) (Figure 5C). *FOXA2* mRNA levels were measured in relation to *SERPINE1* as a verification of TGFβ1/SMAD3 pathway induction. *SERPINE1* level was increased steadily and monotonically by over 2-fold during 24 h stimulation while the *FOXA2* mRNA was repressed at similar level as that observed in A549 cells. The qRT-PCR results in both A549 and human primary SAECs suggested that TGFβ1 modulated mRNA expression of *SERPINE1* and *FOXA2* in pulmonary epithelial cells through SMAD3.

### *FOXA2* Promoter as a Direct Target of SMAD3

**Figure 6:**
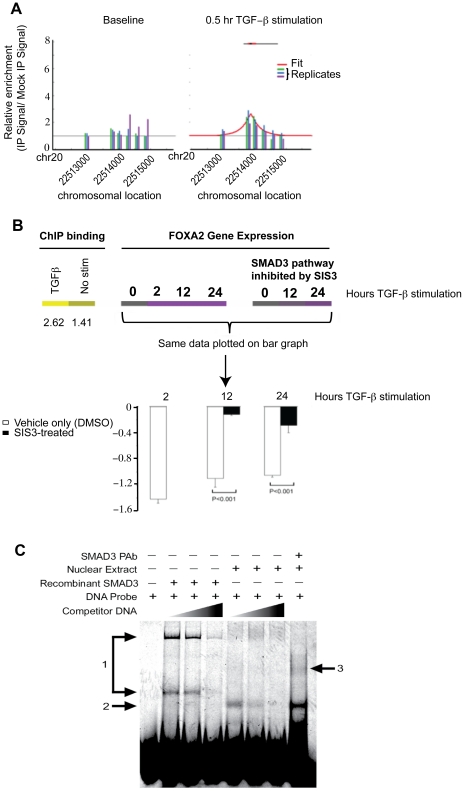
*FOXA2* promoter as a direct target of SMAD3. A: ChIP promoter binding profile of *FOXA2*, baseline (left) and after 30 min 2 ng/ml TGFβ1 stimulation (right). Each bar height indicates respective array signal intensity for that probe. Values from the three promoter array replicates are shown (green, blue, purple, respectively). If the binding was statistically significant, the binding curve (red) is also included and shows the fitted peak shape. B: Heat map illustration specifically of *FOXA2* ChIP binding values (left) with respective gene expression microarray intensities with and without SIS3 treatment (right and far right, respectively). The microarray expression values are plotted in a bar graph (bottom) and show significant repression (white bars) of *FOXA2* during a time course of TGFβ1 treatment that is largely abolished by SIS3 treatment (black bars). C: Electrophoretic mobility shift assay shows specific binding of the SMAD3 protein (lanes 2-4) and nuclear extract from TGFβ1-stimulated A549 cells (lanes 5-7). Lanes 3/6 and 4/7 contain non-labeled competitor *FOXA2* promoter sequence DNA, 40 ng and 200 ng, respectively. Lane 8 contains a polyclonal Ab against SMAD3 and has a supershift band (3).

Since the gene expression study demonstrated *FOXA2* as a novel target of TGFβ1/SMAD3 pathway, we analyzed the direct binding of SMAD3 to its promoter. Significant binding of SMAD3 to *FOXA2* promoter was detected after TGFβ1 stimulation in ChIP-on-chip analysis (Figure 6A). The maximum peak height was 1.41 for basal level and 2.62 after TGFβ1 stimulation. The gene expression of *FOXA2* was reduced by TGFβ1 stimulation and SIS3 significantly abolished this effect at both 6 h and 24 h treatment (Figure 6B). We complemented the ChIP-on chip results by performing EMSA using both SMAD3 recombinant protein and nuclear extract of TGFβ1 stimulated A549 cells and a DNA probe specific for the *FOXA2* promoter (Figure 6C). Specific binding was detected for both recombinant SMAD3 and nuclear extracts. Two protein complexes were identified using recombinant SMAD3 protein (denoted as 1) and competition with unlabeled probes partially abolished both complexes. With nuclear extracts of TGFβ1 stimulated A549 cells, one of these complexes (upper one) was detected in addition to a new protein complex (denoted as 2). Similarly, unlabeled probe competition was able to partially abolish both complexes. The presence of SMAD3 in the complexes associated with nuclear extracts was verified by a supershift analysis using an antibody specific to SMAD3. The supershifted complex was denoted as 3 in Figure 6C. Taken together, we have demonstrated that *FOXA2* promoter was a direct target of SMAD3 protein and its expression was down-regulated by TGFβ1 in pulmonary epithelial cells.

## Discussion

Despite its well-known role as a mediator of TGFβ1 signaling, a comprehensive list of SMAD3 binding targets is not available. To identify SMAD3 binding targets on a genome-wide scale, we performed chromatin immunoprecipitation for SMAD3 in a human lung alveolar epithelial carcinoma cell line A549 and identified its binding targets using promoter region microarrays (ChIP-on-chip). Additionally, a global gene expression analysis was performed in the same cells before and after stimulation with TGFβ1. Analysis of both ChIP-on-chip and gene expression microarray using computational approaches revealed multiple target molecular pathways affected by the TGFβ1/SMAD3 pathway. We have identified a novel TGFβ1/SMAD3 target gene, *FOXA2*, a key regulator of embryonic lung development as well as proper function of the mature lung [50], [51]. Identification of global targets and molecular pathways associated with TGFβ1/SMAD3 pathway will provide insights to its function and lead to better understanding of its important roles in multiple cellular processes.

SMAD3 is a well-known mediator of TGFβ induced-fibrosis. Lack of SMAD3 in mice confers resistance to TGFβ, injury, or inflammation mediated renal and lung fibrosis [52], [53], [54] as well as chemical-induced liver and pancreatic fibrosis [55], [56]. Despite this key role, to the best of our knowledge, this is the first global assessment of SMAD3 targets using ChIP-on-chip technology. Interestingly, genes associated with TGFβ pathway accounted for 10% of directly bounded genes by SMAD3, but many of the pathways affected by TGFβ1/SMAD3 identified using a combination of ChIP-on-Chip and microarray analysis were consistent with the roles of TGFβ in development, fibrosis and cancer. Additionally, multiple known genes associated with EMT and IPF were affected by TGFβ1/SMAD3, including the recently reported *S100A2, RRAS, MYO1D (* Table S3 *)* [40], *SERPINE1* [57] and *TAGLN* [58].

In this study, we identified a novel connection between the TGFβ1/SMAD3 transcriptional regulatory pathway and FOXA2, a transcription factor vitally necessary for lung development and function [50], [59], [60], [61], [62], [63]. TGFβ1 is a known regulator of pulmonary surfactant levels and is known to suppress levels of surfactant protein B (SFTPB) and SFTPC specifically through thyroid transcription factor (TTF-1). Pulmonary surfactants are lipoprotein complexes produced by type II alveolar epithelial cells [64] and play important roles in lung development and normal lung function. Similarly, TTF-1 is also a critical transcription factor in lung development and it is regulated by FOXA2 [65], [66], [67], [68]. Previously it was argued that FOXA2 regulates TTF-1 levels and SFTPB/C through protein-protein interactions [65], [66]. However, the current data strongly suggests that SMAD3 directly binds the promoter of FOXA2 and regulates its activity at the transcriptional level. TGFβ1 selectively activates or represses specific surfactant genes and these regulations are time dependent (data not shown). The exact transcriptional regulatory mechanisms of surfactants through the TGFβ1/SMAD3/FOXA2 regulatory chain remain to be elucidated.

This study provided a comprehensive list of SMAD3 binding targets and global molecular analysis of TGFβ1/SMAD3 signaling networks in the human A549 lung alveolar epithelial cell line. In this context it is important to mention that A549 cells are human alveolar basal epithelial cells derived originally from an explanted adenocarcinoma of the lung. While A549 cells do not necessarily share all features of alveolar epithelial cell, they are commonly used to study pathways and mechanisms relevant to the lung alveolar epithelium because they express alveolar type II markers such as SFTPA2, ZO1 and SFTPC [69], [70], [71]. In our case, we used A549 cells as an *in-vitro* screening tool for identifying specific targets of SMAD3 binding in a lung epithelial cell system. While we believe that the majority of identified SMAD3 target genes in A549 cells are likely to be also true for primary epithelial cells it is plausible that binding targets that require SMAD3 and additional co-factors, only expressed in normal epithelial cells, may not be fully represented in our system. Thus validation of specific TGFβ1/SMAD3 targets in human primary cells is probably needed to focus on specific pathways as we did in the case of *FOXA2* and *SERPINE1*. Naturally, our comprehensive list of SMAD1 targets in A549 cells will be of interest also to cancer researchers because of the role of TGFβ1/SMAD3 signaling in lung cancer [72], [73], [74] and because A549 is also often used in lung cancer research. The analyses of both baseline and after stimulation ChIP-on-chip enhance the mechanistic value of our observations and allow more insights into the pathways recruited in response to TGFβ1/SMAD3 signaling.

In conclusion, the availability of a comprehensive list of SMAD3 signaling targets in response to TGFβ1 stimulation, the analysis of the transcriptional and molecular networks associated with this pathway in lung epithelial cells will improve our understanding of the effects of TGFβ1/SMAD3 signaling in fibrosis and cancer. The discovery of the direct effect of TGFβ1/SMAD3 on FOXA2, a major player in lung development and surfactant production and a key regulator of epithelial cell phenotype, should have significant impact on our understanding of the phenotype of lung alveolar epithelial cells in fibrosis and carcinogenesis and should encourage further research into the role of this molecule in fibrosis.

## Supporting Information

Figure S1 **SMAD3 matrices used for the computational scanning of the SMAD3 binding site.** A: SMAD3 matrix from TRANSFAC (release 2010.1, matrix identifier V$SMAD3_Q6). B and C: SMAD3 matrices from UniPROBE (UniPROBE Accession number UP00000, Smad3_primary and Smad3_secondary). All three SMAD3 matrices were used for the computational scanning. Bound regions were scanned for matrix hits for each of the matrices using a type I cutoff calculated at a *p*-value of 0.05 where the background score distribution was approximated by sampling 10000 instances of the motif length from the uniform distribution and scoring using the motif matrix.
(TIF)

Table S1 **ChIP-on-chip Significant Bound Genes of Non-stimulated A549 Cells.**
(DOCX)

Table S2 **ChIP-on-chip Significant Bound Genes of TGFβ1-stimulated A549 Cells.**
(DOCX)

Table S3 **SMAD3 Target Genes that are Changed in IPF Lungs.**
(DOCX)
