# Supplementary figures and images for: High Throughput Determination of TGFβ1/SMAD3 Targets in A549 Lung Epithelial Cells
Source: PLoS One. 2011 May 20;6(5):e20319. doi: 10.1371/journal.pone.0020319 (PMC3098871; doi:10.1371/journal.pone.0020319)

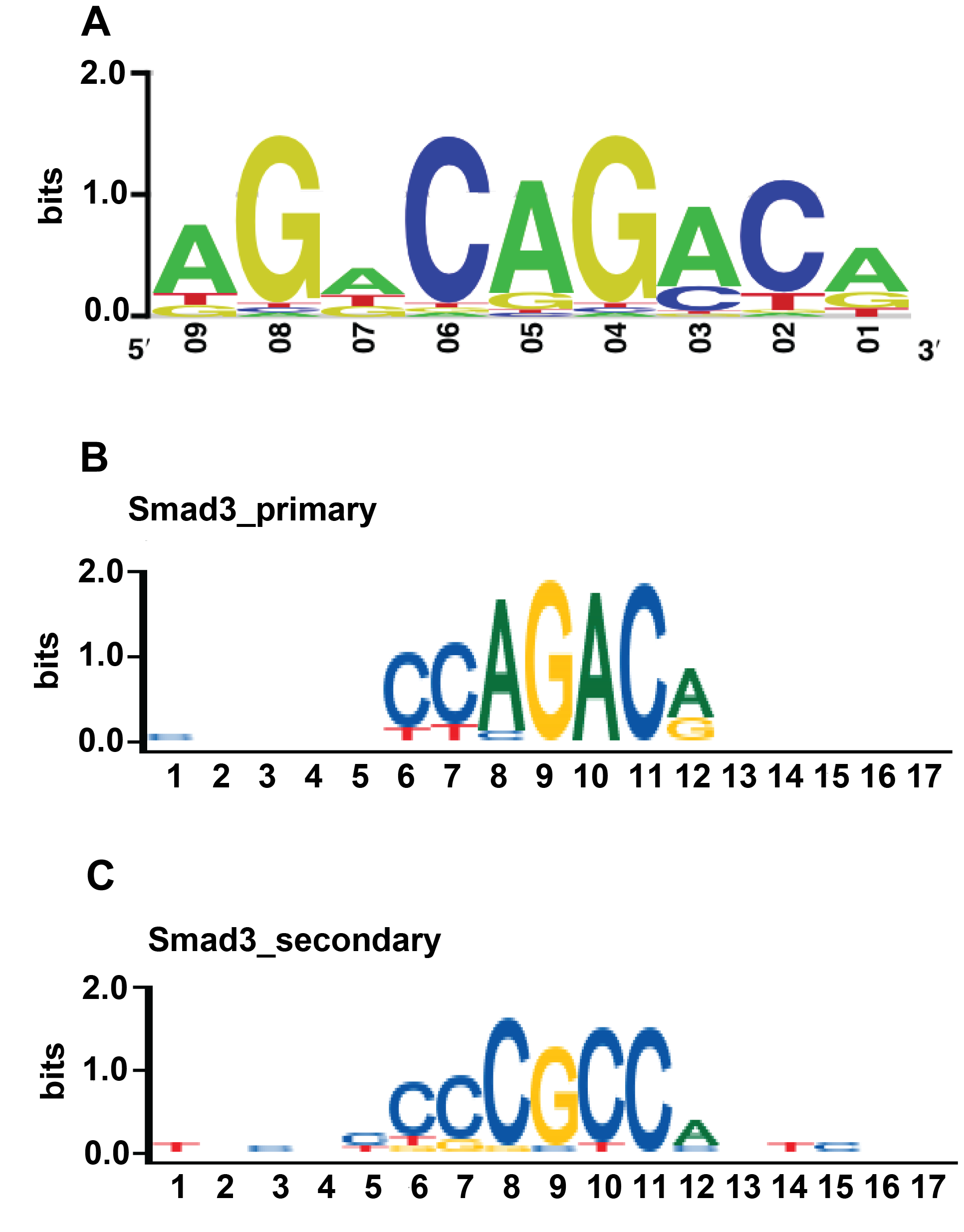

Supplement: Figure S1 — SMAD3 matrices used for the computational scanning of the SMAD3 binding site. A: SMAD3 matrix from TRANSFAC (release 2010.1, matrix identifier V$SMAD3_Q6). B and C: SMAD3 matrices from UniPROBE (UniPROBE Accession number UP00000, Smad3_primary and Smad3_secondary). All three SMAD3 matrices were used for the computational scanning. Bound regions were scanned for matrix hits for each of the matrices using a type I cutoff calculated at a p-value of 0.05 where the background score distribution was approximated by sampling 10000 instances of the motif length from the uniform distribution and scoring using the motif matrix. (TIF) [file pone.0020319.s001.tif]
