# Supplementary material for: High Throughput Determination of TGFβ1/SMAD3 Targets in A549 Lung Epithelial Cells
Source: PLoS One. 2011 May 20;6(5):e20319. doi: 10.1371/journal.pone.0020319 (PMC3098871; doi:10.1371/journal.pone.0020319)
Supplement: Table S1 — ChIP-on-chip Significant Bound Genes of Non-stimulated A549 Cells. (DOCX) [file pone.0020319.s002.docx]

**Table S1. ChIP-on-chip Significant Bound Genes of Non-stimulated A549 Cells**

| Gene ID | Chromosome | Peak Coordinate | Peak Height | Unigene ID | Entrez ID |
| --- | --- | --- | --- | --- | --- |
| NDUFA3 | chr19 | 59297029 | 4.77 | Hs.198269 | 4696 |
| OSCAR | chr19 | 59297029 | 4.77 | Hs.347655 | 126014 |
| SLC16A9 | chr10 | 61166536 | 3.52 | Hs.499709 | 220963 |
| SERPINE1 | chr7 | 100363210 | 3.47 | Hs.414795 | 5054 |
| SRPX2 | chrX | 99698681 | 3.31 | Hs.306339 | 27286 |
| COL7A1 | chr3 | 48608337 | 3.08 | Hs.476218 | 1294 |
| C1orf43 | chr1 | 151010877 | 2.96 | Hs.287471 | 25912 |
| UBAP2L | chr1 | 151010877 | 2.96 | Hs.490551 | 9898 |
| C14orf104 | chr14 | 49173745 | 2.79 | Hs.231761 | 55172 |
| TAAR1 | chr6 | 133009406 | 2.75 | Hs.375030 | 134864 |
| TAP2 | chr6 | 32915843 | 2.73 | Hs.502 | 6891 |
| MTMR7 | chr8 | 17252110 | 2.73 | Hs.625674 | 9108 |
| UGCGL1 | chr2 | 128564724 | 2.69 | Hs.34180 | 56886 |
| SLITRK2 | chrX | 144601633 | 2.6 | Hs.320368 | 84631 |
| CLC | chr11 | 66898625 | 2.57 | Hs.889 | 1178 |
| CD44 | chr11 | 35116202 | 2.5 | Hs.502328 | 960 |
| DDX53 | chrX | 22776995 | 2.46 | Hs.434416 | 168400 |
| FLJ45248 | chr8 | 103888080 | 2.45 | Hs.224506 | 401472 |
| HOXB3 | chr17 | 44007390 | 2.45 | Hs.654560 | 3213 |
| LOXHD1 | chr18 | 42435916 | 2.44 | Hs.345877 | 125336 |
| KIAA1576 | chr16 | 76379759 | 2.44 | Hs.461405 | 57687 |
| PSPC1 | chr13 | 19255871 | 2.43 | Hs.213198 | 55269 |
| SMPX | chrX | 21536243 | 2.42 | Hs.86492 | 23676 |
| HEXB | chr5 | 74015785 | 2.4 | Hs.69293 | 3074 |
| CYP7A1 | chr8 | 59575623 | 2.38 | Hs.1644 | 1581 |
| ZNF45 | chr19 | 49121634 | 2.38 | Hs.381285 | 7596 |
| TEX15 | chr8 | 30826073 | 2.34 | Hs.458316 | 56154 |
| CYP2C8 | chr10 | 96819126 | 2.32 | Hs.282871 | 1558 |
| OR1L3 | chr9 | 122517087 | 2.32 | Hs.626839 | 26735 |
| SMAD6 | chr15 | 64781726 | 2.31 | Hs.153863 | 4091 |
| UBAP2 | chr9 | 34039579 | 2.3 | Hs.493739 | 55833 |
| OR6S1 | chr14 | 20180227 | 2.25 | Hs.513132 | 341799 |
| KIAA1377 | chr11 | 101293218 | 2.24 | Hs.156352 | 57562 |
| ANKRD32 | chr5 | 94039688 | 2.24 | Hs.657315 | 84250 |
| DOCK7 | chr1 | 62774528 | 2.23 | Hs.538059 | 85440 |
| IPO11 | chr5 | 61910383 | 2.21 | Hs.482269 | 51194 |
| C21orf84 | chr21 | 43722480 | 2.21 | Hs.592161 | 114038 |
| MRC1 | chr10 | 17890333 | 2.21 | Hs.75182 | 4360 |
| NR0B1 | chrX | 30088314 | 2.2 | Hs.268490 | 190 |
| TCF20 | chr22 | 40936398 | 2.2 | Hs.475018 | 6942 |
| CHD1 | chr5 | 98290481 | 2.2 | Hs.643465 | 1105 |
| FAM12B | chr14 | 20305919 | 2.19 | Hs.525202 | 64184 |
| SNX12 | chrX | 70071333 | 2.17 | Hs.260750 | 29934 |
| ZNF384 | chr12 | 6668862 | 2.16 | Hs.103315 | 171017 |
| MITF | chr3 | 70068270 | 2.14 | Hs.166017 | 4286 |
| KPTN | chr19 | 52679199 | 2.13 | Hs.25441 | 11133 |
| ITIH1 | chr3 | 52786553 | 2.13 | Hs.420257 | 3697 |
| NEK4 | chr3 | 52786553 | 2.13 | Hs.631921 | 6787 |
| MAGEH1 | chrX | 55361134 | 2.12 | Hs.279819 | 28986 |
| ADPRHL1 | chr13 | 113151705 | 2.12 | Hs.98669 | 113622 |
| C14orf119 | chr14 | 22635264 | 2.1 | Hs.525238 | 55017 |
| PTH | chr11 | 13473995 | 2.09 | Hs.37045 | 5741 |
| POU1F1 | chr3 | 87408136 | 2.09 | Hs.591654 | 5449 |
| MNDA | chr1 | 155613593 | 2.08 | Hs.153837 | 4332 |
| ATAD2 | chr8 | 124478428 | 2.07 | Hs.370834 | 29028 |
| USP9X | chrX | 40738646 | 2.07 | Hs.77578 | 8239 |
| DHX33 | chr17 | 5313841 | 2.06 | Hs.250456 | 56919 |
| WDR47 | chr1 | 109297453 | 2.06 | Hs.654760 | 22911 |
| IFNA8 | chr9 | 21397590 | 2.06 | Hs.73890 | 3445 |
| PLSCR2 | chr3 | 147697009 | 2.05 | Hs.147305 | 57047 |
| RANBP2 | chr2 | 108793750 | 2.05 | Hs.199561 | 5903 |
| C8B | chr1 | 57144784 | 2.05 | Hs.391835 | 732 |
| OR4D10 | chr11 | 59001576 | 2.04 | Hs.553756 | 390197 |
| VNN1 | chr6 | 133077179 | 2.03 | Hs.12114 | 8876 |
| PRDM2 | chr1 | 13846293 | 2.02 | Hs.371823 | 7799 |
| TMPO | chr12 | 97411280 | 2.01 | Hs.11355 | 7112 |
| WNT16 | chr7 | 120558704 | 2.01 | Hs.272375 | 51384 |
| KERA | chr12 | 89954604 | 2 | Hs.125750 | 11081 |
| CDC2 | chr10 | 62113653 | 2 | Hs.334562 | 983 |
| ZNF615 | chr19 | 57187289 | 1.99 | Hs.368355 | 284370 |
| ZNF519 | chr18 | 14143278 | 1.97 | Hs.352635 | 162655 |
| C6orf1 | chr6 | 34325340 | 1.97 | Hs.381300 | 221491 |
| IVD | chr15 | 38484082 | 1.97 | Hs.449599 | 3712 |
| GPHN | chr14 | 66043420 | 1.95 | Hs.208765 | 10243 |
| IL20 | chr1 | 203426897 | 1.95 | Hs.272373 | 50604 |
| CYP39A1 | chr6 | 46727912 | 1.94 | Hs.387367 | 51302 |
| PLCB4 | chr20 | 9024473 | 1.94 | Hs.472101 | 5332 |
| CCDC7 | chr10 | 32896086 | 1.93 | Hs.585464 | 221016 |
| OR7A5 | chr19 | 14800211 | 1.92 | Hs.137573 | 26659 |
| FBXL19 | chr16 | 30867276 | 1.92 | Hs.152149 | 54620 |
| PPM1E | chr17 | 54187524 | 1.92 | Hs.245044 | 22843 |
| NOX3 | chr6 | 155869626 | 1.92 | Hs.247776 | 50508 |
| NQO1 | chr16 | 68318477 | 1.92 | Hs.406515 | 1728 |
| WDR22 | chr14 | 68727664 | 1.92 | Hs.509780 | 8816 |
| SCN10A | chr3 | 38810946 | 1.91 | Hs.250443 | 6336 |
| ZNF146 | chr19 | 41411148 | 1.91 | Hs.643436 | 7705 |
| C10orf120 | chr10 | 124505639 | 1.9 | Hs.363649 | 399814 |
| KDELR2 | chr7 | 6295987 | 1.9 | Hs.654552 | 11014 |
| SGCG | chr13 | 22652816 | 1.89 | Hs.37167 | 6445 |
| ABCG2 | chr4 | 89437822 | 1.89 | Hs.480218 | 9429 |
| OR4A16 | chr11 | 54866702 | 1.89 | Hs.554530 | 81327 |
| CNGB3 | chr8 | 87825115 | 1.88 | Hs.154433 | 54714 |
| SUPT6H | chr17 | 24018952 | 1.87 | Hs.250429 | 6830 |
| RECK | chr9 | 36023213 | 1.87 | Hs.388918 | 8434 |
| RCOR3 | chr1 | 207820743 | 1.87 | Hs.696152 | 55758 |
| GJA1 | chr6 | 121798652 | 1.87 | Hs.74471 | 2697 |
| DLAT | chr11 | 111400951 | 1.86 | Hs.335551 | 1737 |
| DBP | chr19 | 53832851 | 1.86 | Hs.414480 | 1628 |
| OCM | chr7 | 97264605 | 1.86 | Hs.571315 | 654231 |
| SUMF2 | chr7 | 55905471 | 1.85 | Hs.279696 | 25870 |
| CCT6A | chr7 | 55905471 | 1.85 | Hs.82916 | 908 |
| NME1 | chr17 | 46586011 | 1.84 | Hs.463456 | 4830 |
| MGAT5 | chr2 | 134842456 | 1.84 | Hs.651869 | 4249 |
| ARHGAP1 | chr11 | 46678958 | 1.83 | Hs.138860 | 392 |
| GPNMB | chr7 | 23059819 | 1.83 | Hs.190495 | 10457 |
| PDZRN4 | chr12 | 40117191 | 1.83 | Hs.380044 | 29951 |
| ZNF408 | chr11 | 46678958 | 1.83 | Hs.656931 | 79797 |
| SLC31A2 | chr9 | 112992311 | 1.82 | Hs.24030 | 1318 |
| WFDC3 | chr20 | 43853533 | 1.82 | Hs.419126 | 140686 |
| SCEL | chr13 | 77007485 | 1.81 | Hs.534699 | 8796 |
| DMTF1 | chr7 | 86425809 | 1.81 | Hs.654981 | 9988 |
| ADH4 | chr4 | 100423498 | 1.8 | Hs.1219 | 127 |
| DDX5 | chr17 | 59933430 | 1.8 | Hs.279806 | 1655 |
| P4HA2 | chr5 | 131594940 | 1.8 | Hs.519568 | 8974 |
| OR4D9 | chr11 | 59038961 | 1.8 | Hs.553757 | 390199 |
| TMEM20 | chr10 | 95643115 | 1.8 | Hs.632085 | 159371 |
| CDC14A | chr1 | 100529425 | 1.79 | Hs.127411 | 8556 |
| COTL1 | chr16 | 83210100 | 1.79 | Hs.289092 | 23406 |
| RAB11A | chr15 | 63948553 | 1.79 | Hs.321541 | 8766 |
| S100A8 | chr1 | 150177350 | 1.79 | Hs.416073 | 6279 |
| MRPL39 | chr21 | 25901604 | 1.79 | Hs.420696 | 54148 |
| MCM7 | chr7 | 99342378 | 1.79 | Hs.438720 | 4176 |
| ULK1 | chr12 | 131044985 | 1.79 | Hs.47061 | 8408 |
| MORF4L2 | chrX | 102749697 | 1.78 | Hs.326387 | 9643 |
| SMAD7 | chr18 | 44733137 | 1.78 | Hs.465087 | 4092 |
| CSNK1E | chr22 | 37038496 | 1.78 | Hs.474833 | 1454 |
| C6orf170 | chr6 | 121700992 | 1.77 | Hs.121396 | 221322 |
| FOXA3 | chr19 | 51061205 | 1.77 | Hs.36137 | 3171 |
| LCP1 | chr13 | 45654615 | 1.77 | Hs.381099 | 3936 |
| TRIB1 | chr8 | 126511550 | 1.77 | Hs.444947 | 10221 |
| GRM8 | chr7 | 126477011 | 1.77 | Hs.449625 | 2918 |
| FILIP1 | chr6 | 76260824 | 1.77 | Hs.696158 | 27145 |
| RAB2B | chr14 | 21015028 | 1.76 | Hs.22399 | 84932 |
| SLC19A2 | chr1 | 166187466 | 1.76 | Hs.30246 | 10560 |
| LECT2 | chr5 | 135318727 | 1.76 | Hs.512580 | 3950 |
| KCNJ5 | chr11 | 128280830 | 1.76 | Hs.632109 | 3762 |
| SCN2B | chr11 | 117553038 | 1.75 | Hs.129783 | 6327 |
| MBIP | chr14 | 35860164 | 1.75 | Hs.368647 | 51562 |
| C14orf124 | chr14 | 23982004 | 1.75 | Hs.643552 | 56948 |
| RPH3AL | chr17 | 202894 | 1.75 | Hs.651925 | 9501 |
| NDUFB2 | chr7 | 139849701 | 1.75 | Hs.655788 | 4708 |
| TRIM16 | chr17 | 15486944 | 1.74 | Hs.123534 | 10626 |
| XDH | chr2 | 31549147 | 1.74 | Hs.250 | 7498 |
| PSMC3 | chr11 | 47404463 | 1.74 | Hs.250758 | 5702 |
| SFTPD | chr10 | 81698500 | 1.74 | Hs.253495 | 6441 |
| RAB1A | chr2 | 65269496 | 1.74 | Hs.310645 | 5861 |
| CLDN11 | chr3 | 171618867 | 1.74 | Hs.31595 | 5010 |
| HDHD2 | chr18 | 42917339 | 1.74 | Hs.465041 | 84064 |
| OR8H3 | chr11 | 55646721 | 1.74 | Hs.553745 | 390152 |
| MLL5 | chr7 | 104248288 | 1.74 | Hs.592262 | 55904 |
| TBL1X | chrX | 9240896 | 1.74 | Hs.699315 | 6907 |
| UMPS | chr3 | 125931372 | 1.73 | Hs.2057 | 7372 |
| USP52 | chr12 | 55014107 | 1.73 | Hs.273397 | 9924 |
| RPS4Y1 | chrY | 2753385 | 1.73 | Hs.282376 | 6192 |
| ENTPD1 | chr10 | 97506244 | 1.73 | Hs.576612 | 953 |
| CALM2 | chr2 | 47315534 | 1.73 | Hs.643483 | 805 |
| GNRHR | chr4 | 68450418 | 1.72 | Hs.407587 | 2798 |
| POLI | chr18 | 50046745 | 1.72 | Hs.438533 | 11201 |
| OPA3 | chr19 | 50779847 | 1.72 | Hs.466945 | 80207 |
| FBXL2 | chr3 | 33293457 | 1.72 | Hs.475872 | 25827 |
| MAGEA9 | chrX | 148568579 | 1.72 | Hs.512582 | 4108 |
| PJA1 | chrX | 68171297 | 1.72 | Hs.522679 | 64219 |
| SUMF1 | chr3 | 4486305 | 1.72 | Hs.588682 | 285362 |
| ZDHHC7 | chr16 | 83603064 | 1.72 | Hs.592065 | 55625 |
| TMEM41A | chr3 | 186699766 | 1.72 | Hs.634586 | 90407 |
| AKR1C3 | chr10 | 5126801 | 1.72 | Hs.78183 | 8644 |
| ARID4A | chr14 | 57834609 | 1.71 | Hs.161000 | 5926 |
| NAP1L3 | chrX | 92734518 | 1.71 | Hs.21365 | 4675 |
| WWP2 | chr16 | 68347093 | 1.71 | Hs.408458 | 11060 |
| IPO4 | chr14 | 23727940 | 1.71 | Hs.411865 | 79711 |
| RRAS | chr19 | 54835840 | 1.71 | Hs.515536 | 6237 |
| HFE2 | chr1 | 142901919 | 1.71 | Hs.632436 | 148738 |
| POM121 | chr7 | 71794157 | 1.71 | Hs.655217 | 9883 |
| VASP | chr19 | 50702309 | 1.71 | Hs.702197 | 7408 |
| PSMB7 | chr9 | 124257544 | 1.7 | Hs.213470 | 5695 |
| ART3 | chr4 | 77352442 | 1.7 | Hs.24976 | 419 |
| IL13RA2 | chrX | 114075588 | 1.7 | Hs.336046 | 3598 |
| SLC22A9 | chr11 | 62893353 | 1.7 | Hs.502772 | 114571 |
| ZNF410 | chr14 | 73423251 | 1.69 | Hs.270869 | 57862 |
| PIAS3 | chr1 | 143065373 | 1.69 | Hs.435761 | 10401 |
| TPSG1 | chr16 | 1299302 | 1.69 | Hs.592076 | 25823 |
| PPM1A | chr14 | 59783361 | 1.69 | Hs.592298 | 5494 |
| ZBTB1 | chr14 | 64041004 | 1.69 | Hs.655536 | 22890 |
| HNRPUL1 | chr19 | 46460268 | 1.69 | Hs.699274 | 11100 |
| SLITRK4 | chrX | 142450168 | 1.68 | Hs.272284 | 139065 |
| USP3 | chr15 | 61583755 | 1.68 | Hs.458499 | 9960 |
| HSPA5 | chr9 | 125083587 | 1.68 | Hs.605502 | 3309 |
| GABRA3 | chrX | 151291028 | 1.67 | Hs.123024 | 2556 |
| USP32 | chr17 | 55824637 | 1.67 | Hs.132868 | 84669 |
| MRCL3 | chr18 | 3242386 | 1.67 | Hs.190086 | 10627 |
| IL22 | chr12 | 66934123 | 1.67 | Hs.287369 | 50616 |
| C5orf15 | chr5 | 133332701 | 1.67 | Hs.355177 | 56951 |
| SSX6 | chrX | 47722615 | 1.67 | Hs.511998 | 280657 |
| PON2 | chr7 | 94708867 | 1.67 | Hs.530077 | 5445 |
| TOB1 | chr17 | 46296731 | 1.67 | Hs.531550 | 10140 |
| CCDC9 | chr19 | 52451786 | 1.66 | Hs.227782 | 26093 |
| WDR34 | chr9 | 128499213 | 1.66 | Hs.495240 | 89891 |
| BRMS1L | chr14 | 35365290 | 1.66 | Hs.525299 | 84312 |
| PTPN22 | chr1 | 114126730 | 1.66 | Hs.535276 | 26191 |
| NCOA5 | chr20 | 44152362 | 1.66 | Hs.654991 | 57727 |
| PIN4 | chrX | 71184286 | 1.66 | Hs.655623 | 5303 |
| SMYD2 | chr1 | 210842355 | 1.66 | Hs.66170 | 56950 |
| EDG6 | chr19 | 3128063 | 1.66 | Hs.662006 | 8698 |
| CSN1S1 | chr4 | 70977652 | 1.65 | Hs.3155 | 1446 |
| CPA2 | chr7 | 129500143 | 1.65 | Hs.490038 | 1358 |
| SEMA3E | chr7 | 82922708 | 1.65 | Hs.528721 | 9723 |
| OR1S2 | chr11 | 57728782 | 1.65 | Hs.553644 | 219958 |
| OR1S1 | chr11 | 57728782 | 1.65 | Hs.553645 | 219959 |
| PRKAB1 | chr12 | 118568560 | 1.65 | Hs.6061 | 5564 |
| DHX34 | chr19 | 52544671 | 1.64 | Hs.151706 | 9704 |
| DMC1 | chr22 | 37291160 | 1.64 | Hs.339396 | 11144 |
| EXTL2 | chr1 | 101073902 | 1.64 | Hs.357637 | 2135 |
| ITGB6 | chr2 | 160881999 | 1.64 | Hs.470399 | 3694 |
| SLC30A7 | chr1 | 101073902 | 1.64 | Hs.533903 | 148867 |
| C5orf5 | chr5 | 137399346 | 1.64 | Hs.657919 | 51306 |
| USP51 | chrX | 55398778 | 1.63 | Hs.134289 | 158880 |
| FMO2 | chr1 | 167886496 | 1.63 | Hs.144912 | 2327 |
| HDC | chr15 | 48345821 | 1.63 | Hs.1481 | 3067 |
| C14orf4 | chr14 | 76564957 | 1.63 | Hs.179260 | 64207 |
| GSR | chr8 | 30705256 | 1.63 | Hs.271510 | 2936 |
| PDGFD | chr11 | 103412055 | 1.63 | Hs.352298 | 80310 |
| BEX2 | chrX | 102372515 | 1.63 | Hs.398989 | 84707 |
| CDC14B | chr9 | 96497618 | 1.63 | Hs.40582 | 8555 |
| KIAA0831 | chr14 | 54948552 | 1.63 | Hs.414809 | 22863 |
| CHM | chrX | 85108452 | 1.63 | Hs.496449 | 1121 |
| KCTD7 | chr7 | 65537356 | 1.63 | Hs.546627 | 154881 |
| OR2T12 | chr1 | 244784752 | 1.63 | Hs.553582 | 127064 |
| SLC23A1 | chr5 | 138747088 | 1.63 | Hs.643467 | 9963 |
| GNAS | chr20 | 56847652 | 1.62 | Hs.125898 | 2778 |
| MAN2C1 | chr15 | 73447968 | 1.62 | Hs.26232 | 4123 |
| PRRG2 | chr19 | 54775663 | 1.62 | Hs.35101 | 5639 |
| PRKAB2 | chr1 | 143869058 | 1.62 | Hs.50732 | 5565 |
| OCIAD1 | chr4 | 48674154 | 1.62 | Hs.518750 | 54940 |
| FH | chr1 | 238009637 | 1.62 | Hs.592490 | 2271 |
| DNAH10 | chr12 | 122844972 | 1.62 | Hs.622654 | 196385 |
| PDC | chr1 | 183162803 | 1.62 | Hs.654381 | 5132 |
| NOSIP | chr19 | 54775663 | 1.62 | Hs.7236 | 51070 |
| CHRNA6 | chr8 | 42743413 | 1.61 | Hs.103128 | 8973 |
| UVRAG | chr11 | 75203832 | 1.61 | Hs.202470 | 7405 |
| CCL14 | chr17 | 31338727 | 1.61 | Hs.272493 | 6358 |
| NPHP1 | chr2 | 110319642 | 1.61 | Hs.280388 | 4867 |
| LBR | chr1 | 221922959 | 1.61 | Hs.435166 | 3930 |
| SUV39H1 | chrX | 48311058 | 1.61 | Hs.522639 | 6839 |
| OR4M1 | chr14 | 19297338 | 1.61 | Hs.553829 | 441670 |
| MYEF2 | chr15 | 46257844 | 1.61 | Hs.6638 | 50804 |
| NDUFS2 | chr1 | 157996492 | 1.6 | Hs.173611 | 4720 |
| PARP2 | chr14 | 19881928 | 1.6 | Hs.409412 | 10038 |
| LOC51057 | chr2 | 63727813 | 1.6 | Hs.414952 | 51057 |
| CYHR1 | chr8 | 145661936 | 1.6 | Hs.459379 | 50626 |
| MDH1 | chr2 | 63727813 | 1.6 | Hs.526521 | 4190 |
| OR4K13 | chr14 | 19572801 | 1.6 | Hs.553573 | 390433 |
| FUCA2 | chr6 | 143874430 | 1.6 | Hs.591332 | 2519 |
| SERPINA12 | chr14 | 94054271 | 1.6 | Hs.99476 | 145264 |
| SMAD2 | chr18 | 43711910 | 1.59 | Hs.12253 | 4087 |
| NEGR1 | chr1 | 72143388 | 1.59 | Hs.146542 | 257194 |
| H3F3B | chr17 | 71292757 | 1.59 | Hs.180877 | 3021 |
| ZNF302 | chr19 | 39860094 | 1.59 | Hs.436350 | 55900 |
| TWSG1 | chr18 | 9324233 | 1.59 | Hs.514685 | 57045 |
| SERPINA1 | chr14 | 93925072 | 1.59 | Hs.525557 | 5265 |
| CASP10 | chr2 | 201873601 | 1.59 | Hs.5353 | 843 |
| ASB5 | chr4 | 177565934 | 1.59 | Hs.591712 | 140458 |
| B2M | chr15 | 42791021 | 1.59 | Hs.626605 | 567 |
| KIF23 | chr15 | 67493347 | 1.58 | Hs.270845 | 9493 |
| DCTN4 | chr5 | 150118562 | 1.58 | Hs.328865 | 51164 |
| PPIG | chr2 | 170266415 | 1.58 | Hs.470544 | 9360 |
| AQR | chr15 | 33049385 | 1.58 | Hs.510958 | 9716 |
| AP2B1 | chr17 | 30938509 | 1.58 | Hs.514819 | 163 |
| PEX12 | chr17 | 30938509 | 1.58 | Hs.591190 | 5193 |
| LRRC6 | chr8 | 133757427 | 1.58 | Hs.591865 | 23639 |
| LPPR2 | chr19 | 11326982 | 1.58 | Hs.6846 | 64748 |
| FAH | chr15 | 78232448 | 1.58 | Hs.73875 | 2184 |
| ZBTB3 | chr11 | 62278130 | 1.57 | Hs.147554 | 79842 |
| RHOBTB2 | chr8 | 22900429 | 1.57 | Hs.372688 | 23221 |
| PEBP4 | chr8 | 22900429 | 1.57 | Hs.491242 | 157310 |
| GJB2 | chr13 | 19665586 | 1.57 | Hs.524894 | 2706 |
| JMJD1A | chr2 | 86579936 | 1.57 | Hs.557425 | 55818 |
| NCOA1 | chr2 | 24719085 | 1.57 | Hs.699183 | 8648 |
| SLC39A6 | chr18 | 31963764 | 1.57 | Hs.79136 | 25800 |
| HTR1E | chr6 | 87703528 | 1.56 | Hs.1611 | 3354 |
| PPP1CA | chr11 | 66925930 | 1.56 | Hs.183994 | 5499 |
| KIAA0528 | chr12 | 22588600 | 1.56 | Hs.271014 | 9847 |
| TSGA14 | chr7 | 129674683 | 1.56 | Hs.368315 | 95681 |
| FLJ34503 | chr6 | 114332086 | 1.56 | Hs.376634 | 285759 |
| EFHA1 | chr13 | 21076460 | 1.56 | Hs.412103 | 221154 |
| NUDCD3 | chr7 | 44303532 | 1.56 | Hs.488171 | 23386 |
| OR2T29 | chr1 | 245049053 | 1.56 | Hs.553707 | 343563 |
| PCDH9 | chr13 | 66702134 | 1.56 | Hs.654709 | 5101 |
| BNC2 | chr9 | 16518145 | 1.56 | Hs.656581 | 54796 |
| AKAP10 | chr17 | 19821894 | 1.56 | Hs.694769 | 11216 |
| FOXP4 | chr6 | 41621769 | 1.55 | Hs.131436 | 116113 |
| SENP1 | chr12 | 46786121 | 1.55 | Hs.371957 | 29843 |
| MAP2K6 | chr17 | 64921720 | 1.55 | Hs.463978 | 5608 |
| SLC9A11 | chr1 | 170303808 | 1.55 | Hs.494981 | 284525 |
| ZNF41 | chrX | 47099473 | 1.55 | Hs.496074 | 7592 |
| SPRED1 | chr15 | 36331390 | 1.55 | Hs.525781 | 161742 |
| GSPT1 | chr16 | 11917888 | 1.55 | Hs.528780 | 2935 |
| COPS7A | chr12 | 6703670 | 1.55 | Hs.530823 | 50813 |
| SCAP | chr3 | 47460018 | 1.55 | Hs.531789 | 22937 |
| RIN1 | chr11 | 65860893 | 1.54 | Hs.1030 | 9610 |
| C14orf126 | chr14 | 30997058 | 1.54 | Hs.116014 | 112487 |
| EPHX2 | chr8 | 27404909 | 1.54 | Hs.212088 | 2053 |
| NFYC | chr1 | 40826336 | 1.54 | Hs.233458 | 4802 |
| C21orf81 | chr21 | 14274500 | 1.54 | Hs.364456 | 114035 |
| RBJ | chr2 | 25106231 | 1.54 | Hs.434993 | 51277 |
| FAM33A | chr17 | 54587580 | 1.54 | Hs.463607 | 348235 |
| MTF1 | chr1 | 37995050 | 1.54 | Hs.471991 | 4520 |
| GNL3L | chrX | 54439498 | 1.54 | Hs.654677 | 54552 |
| SNX11 | chr17 | 43533441 | 1.53 | Hs.15827 | 29916 |
| PRPF18 | chr10 | 13669044 | 1.53 | Hs.161181 | 8559 |
| ARIH1 | chr15 | 70553133 | 1.53 | Hs.268787 | 25820 |
| TXN | chr9 | 110098898 | 1.53 | Hs.435136 | 7295 |
| NEUROD1 | chr2 | 182371225 | 1.53 | Hs.440955 | 4760 |
| GLT6D1 | chr9 | 135757344 | 1.53 | Hs.522491 | 360203 |
| NOTCH2NL | chr1 | 142698018 | 1.53 | Hs.655156 | 388677 |
| C14orf43 | chr14 | 73297039 | 1.53 | Hs.656506 | 91748 |
| CBX1 | chr17 | 43533441 | 1.53 | Hs.77254 | 10951 |
| HSPE1 | chr2 | 198190437 | 1.52 | Hs.1197 | 3336 |
| PARP15 | chr3 | 123817017 | 1.52 | Hs.120250 | 165631 |
| HAP1 | chr17 | 37144782 | 1.52 | Hs.158300 | 9001 |
| TAF1 | chrX | 70368810 | 1.52 | Hs.158560 | 6872 |
| CASP9 | chr1 | 15596649 | 1.52 | Hs.329502 | 842 |
| HIST4H4 | chr12 | 14815460 | 1.52 | Hs.352191 | 121504 |
| RPS17 | chr15 | 81006712 | 1.52 | Hs.433427 | 6218 |
| DAB2 | chr5 | 39461357 | 1.52 | Hs.481980 | 1601 |
| RUSC2 | chr9 | 35480236 | 1.52 | Hs.493796 | 9853 |
| TAGLN2 | chr1 | 156708123 | 1.52 | Hs.517168 | 8407 |
| EPS8 | chr12 | 15833878 | 1.52 | Hs.591160 | 2059 |
| HSPD1 | chr2 | 198190437 | 1.52 | Hs.595053 | 3329 |
| LOX | chr5 | 121442444 | 1.51 | Hs.102267 | 4015 |
| UQCRB | chr8 | 97316911 | 1.51 | Hs.131255 | 7381 |
| ACAA2 | chr18 | 45594422 | 1.51 | Hs.200136 | 10449 |
| STMN4 | chr8 | 27172484 | 1.51 | Hs.201058 | 81551 |
| FLJ11184 | chr4 | 164773158 | 1.51 | Hs.267446 | 55319 |
| MAP3K3 | chr17 | 59053487 | 1.51 | Hs.29282 | 4215 |
| FEN1 | chr11 | 61316809 | 1.51 | Hs.409065 | 2237 |
| TARS | chr5 | 33476771 | 1.51 | Hs.481860 | 6897 |
| ATXN3 | chr14 | 91642634 | 1.51 | Hs.532632 | 4287 |
| DLG7 | chr14 | 54728024 | 1.51 | Hs.77695 | 9787 |
| SHOC2 | chr10 | 112669455 | 1.5 | Hs.104315 | 8036 |
| PTDSS2 | chr11 | 440101 | 1.5 | Hs.12851 | 81490 |
| AQP2 | chr12 | 48626142 | 1.5 | Hs.130730 | 359 |
| SNAP25 | chr20 | 10147254 | 1.5 | Hs.167317 | 6616 |
| EHBP1 | chr2 | 62844729 | 1.5 | Hs.271667 | 23301 |
| RBBP8 | chr18 | 18767002 | 1.5 | Hs.546282 | 5932 |
| SREBF1 | chr17 | 17681583 | 1.5 | Hs.592123 | 6720 |
| HEXA | chr15 | 70455622 | 1.5 | Hs.604479 | 3073 |
| MANBAL | chr20 | 35351340 | 1.5 | Hs.6126 | 63905 |
| ZBTB20 | chr3 | 116348890 | 1.5 | Hs.655108 | 26137 |
| NEK11 | chr3 | 132229027 | 1.5 | Hs.657336 | 79858 |
| PPP1R11 | chr6 | 30142911 | 1.5 | Hs.82887 | 6992 |
