# Supplementary material for: High Throughput Determination of TGFβ1/SMAD3 Targets in A549 Lung Epithelial Cells
Source: PLoS One. 2011 May 20;6(5):e20319. doi: 10.1371/journal.pone.0020319 (PMC3098871; doi:10.1371/journal.pone.0020319)
Supplement: Table S2 — ChIP-on-chip Significant Bound Genes of TGFβ1-stimulated A549 Cells. (DOCX) [file pone.0020319.s003.docx]

Table S2. ChIP-on-chip Significant Bound Genes of TGFβ1-stimulated A549 Cells

| Gene ID | Chromosome | Peak Coordinate | Peak Height | Unigene ID | Entrez ID |
| --- | --- | --- | --- | --- | --- |
| SERPINE1 | chr7 | 100363260 | 10.4 | Hs.414795 | 5054 |
| FLJ45248 | chr8 | 103888380 | 7.67 | Hs.224506 | 401472 |
| CCDC129 | chr7 | 31329585 | 7.5 | Hs.224269 | 223075 |
| CLC | chr11 | 66898250 | 6.62 | Hs.889 | 1178 |
| PPP1R13L | chr19 | 50600927 | 6.08 | Hs.466937 | 10848 |
| COL7A1 | chr3 | 48608364 | 4.63 | Hs.476218 | 1294 |
| SMAD6 | chr15 | 64781701 | 4.63 | Hs.153863 | 4091 |
| STXBP1 | chr9 | 127420790 | 4.2 | Hs.288229 | 6812 |
| FAM129B | chr9 | 127420790 | 4.2 | Hs.522401 | 64855 |
| FGB | chr4 | 155841477 | 4.1 | Hs.300774 | 2244 |
| TINAGL1 | chr1 | 31710449 | 4 | Hs.199368 | 64129 |
| POLD4 | chr11 | 66877802 | 3.98 | Hs.523829 | 57804 |
| SMAD7 | chr18 | 44733037 | 3.91 | Hs.465087 | 4092 |
| BCL9L | chr11 | 118286894 | 3.84 | Hs.414740 | 283149 |
| GRIN2D | chr19 | 53589601 | 3.76 | Hs.445015 | 2906 |
| KDELR1 | chr19 | 53589601 | 3.76 | Hs.515515 | 10945 |
| TAGLN2 | chr1 | 156708173 | 3.72 | Hs.517168 | 8407 |
| MRC2 | chr17 | 58058491 | 3.72 | Hs.7835 | 9902 |
| RUSC2 | chr9 | 35480336 | 3.7 | Hs.493796 | 9853 |
| MUC1 | chr1 | 151912426 | 3.68 | Hs.89603 | 4582 |
| DOCK7 | chr1 | 62775228 | 3.6 | Hs.538059 | 85440 |
| SRXN1 | chr20 | 582284 | 3.51 | Hs.355284 | 140809 |
| SNX12 | chrX | 70071283 | 3.34 | Hs.260750 | 29934 |
| S100A2 | chr1 | 150352089 | 3.31 | Hs.516484 | 6273 |
| RRAS | chr19 | 54835826 | 3.3 | Hs.515536 | 6237 |
| BLOC1S2 | chr10 | 102036369 | 3.3 | Hs.702055 | 282991 |
| PINX1 | chr8 | 10735944 | 3.27 | Hs.490991 | 54984 |
| MYO1D | chr17 | 28228306 | 3.23 | Hs.658000 | 4642 |
| FLJ10357 | chr14 | 20608299 | 3.2 | Hs.35125 | 55701 |
| TRIB1 | chr8 | 126511625 | 3.17 | Hs.444947 | 10221 |
| C21orf84 | chr21 | 43722455 | 3.04 | Hs.592161 | 114038 |
| GIPC1 | chr19 | 14468223 | 3.03 | Hs.655012 | 10755 |
| ACLY | chr17 | 37329292 | 3.01 | Hs.387567 | 47 |
| ENO3 | chr17 | 4793679 | 2.99 | Hs.224171 | 2027 |
| PFN1 | chr17 | 4793679 | 2.99 | Hs.494691 | 5216 |
| IFI44 | chr1 | 78827203 | 2.97 | Hs.82316 | 10561 |
| VASP | chr19 | 50701959 | 2.95 | Hs.702197 | 7408 |
| DC2 | chr4 | 109928502 | 2.95 | Hs.445803 | 58505 |
| ITGA3 | chr17 | 45488214 | 2.93 | Hs.265829 | 3675 |
| PALLD | chr4 | 169792289 | 2.92 | Hs.151220 | 23022 |
| UPP1 | chr7 | 47901807 | 2.9 | Hs.488240 | 7378 |
| HNRPUL1 | chr19 | 46460343 | 2.9 | Hs.699274 | 11100 |
| ACYP2 | chr2 | 54253637 | 2.89 | Hs.516173 | 98 |
| NXNL2 | chr9 | 88379337 | 2.83 | Hs.668937 | 158046 |
| EPS8 | chr12 | 15833903 | 2.78 | Hs.591160 | 2059 |
| RCOR3 | chr1 | 207821068 | 2.77 | Hs.696152 | 55758 |
| CS | chr12 | 54981195 | 2.75 | Hs.430606 | 1431 |
| C11orf1 | chr11 | 111255208 | 2.74 | Hs.17546 | 64776 |
| ALG9 | chr11 | 111255208 | 2.74 | Hs.503850 | 79796 |
| ZYX | chr7 | 142595083 | 2.71 | Hs.490415 | 7791 |
| HERPUD2 | chr7 | 35507913 | 2.7 | Hs.599851 | 64224 |
| IL31RA | chr5 | 55185041 | 2.7 | Hs.55378 | 133396 |
| TGFB1 | chr19 | 46551433 | 2.69 | Hs.645227 | 7040 |
| SLC20A1 | chr2 | 113119310 | 2.68 | Hs.187946 | 6574 |
| QPCT | chr2 | 37483722 | 2.67 | Hs.79033 | 25797 |
| PRKAB2 | chr1 | 143869008 | 2.66 | Hs.50732 | 5565 |
| C14orf79 | chr14 | 104508001 | 2.66 | Hs.27183 | 122616 |
| CALM2 | chr2 | 47315509 | 2.62 | Hs.643483 | 805 |
| FOXA2 | chr20 | 22513876 | 2.62 | Hs.155651 | 3170 |
| TAGLN | chr11 | 116575232 | 2.62 | Hs.632099 | 6876 |
| C14orf4 | chr14 | 76564957 | 2.62 | Hs.179260 | 64207 |
| NAB2 | chr12 | 55768710 | 2.6 | Hs.159223 | 4665 |
| TMEM194 | chr12 | 55768710 | 2.6 | Hs.591040 | 23306 |
| ATAD2 | chr8 | 124478103 | 2.6 | Hs.370834 | 29028 |
| C14orf43 | chr14 | 73296989 | 2.6 | Hs.656506 | 91748 |
| IL11 | chr19 | 60573762 | 2.59 | Hs.467304 | 3589 |
| TMEM190 | chr19 | 60573762 | 2.59 | Hs.590943 | 147744 |
| SMAD3 | chr15 | 65145141 | 2.58 | Hs.618504 | 4088 |
| USP3 | chr15 | 61583805 | 2.56 | Hs.458499 | 9960 |
| WDR55 | chr5 | 140024137 | 2.55 | Hs.286261 | 54853 |
| SH2D4A | chr8 | 19215477 | 2.54 | Hs.303208 | 63898 |
| KIF21A | chr12 | 38123694 | 2.53 | Hs.374201 | 55605 |
| ARF6 | chr14 | 49429281 | 2.52 | Hs.525330 | 382 |
| BAIAP2L1 | chr7 | 97676096 | 2.52 | Hs.656063 | 55971 |
| PDE7B | chr6 | 136213988 | 2.51 | Hs.652367 | 27115 |
| GTF2B | chr1 | 89069580 | 2.5 | Hs.481852 | 2959 |
| ITGB1 | chr10 | 33287565 | 2.5 | Hs.695946 | 3688 |
| GCC2 | chr2 | 108524118 | 2.48 | Hs.705434 | 9648 |
| ZCCHC11 | chr1 | 52671855 | 2.48 | Hs.655407 | 23318 |
| SPRED1 | chr15 | 36331415 | 2.48 | Hs.525781 | 161742 |
| ANXA2 | chr15 | 58477409 | 2.47 | Hs.511605 | 302 |
| PLAUR | chr19 | 48866577 | 2.47 | Hs.466871 | 5329 |
| PLS3 | chrX | 114618611 | 2.47 | Hs.496622 | 5358 |
| SEMA4B | chr15 | 88529216 | 2.47 | Hs.474935 | 10509 |
| ZNF219 | chr14 | 20645408 | 2.46 | Hs.250493 | 51222 |
| C14orf104 | chr14 | 49173738 | 2.44 | Hs.231761 | 55172 |
| C14orf24 | chr14 | 34585341 | 2.44 | Hs.446357 | 283635 |
| KPNA1 | chr3 | 123717286 | 2.41 | Hs.161008 | 3836 |
| SOX1 | chr13 | 111770146 | 2.4 | Hs.202526 | 6656 |
| SULT2B1 | chr19 | 53747103 | 2.4 | Hs.369331 | 6820 |
| TRIM16 | chr17 | 15486869 | 2.36 | Hs.123534 | 10626 |
| CA3 | chr8 | 86537250 | 2.35 | Hs.82129 | 761 |
| CSN2 | chr4 | 71007212 | 2.35 | Hs.2242 | 1447 |
| DDX5 | chr17 | 59933430 | 2.35 | Hs.279806 | 1655 |
| CCDC45 | chr17 | 59933430 | 2.35 | Hs.569713 | 90799 |
| PVR | chr19 | 49839000 | 2.33 | Hs.171844 | 5817 |
| XRCC2 | chr7 | 151811527 | 2.33 | Hs.647093 | 7516 |
| MEX3B | chr15 | 80125763 | 2.33 | Hs.104744 | 84206 |
| UTRN | chr6 | 144654729 | 2.32 | Hs.133135 | 7402 |
| DHRS12 | chr13 | 51276108 | 2.32 | Hs.266728 | 79758 |
| JMJD2A | chr1 | 43785036 | 2.31 | Hs.155983 | 9682 |
| ZBTB45 | chr19 | 63723242 | 2.31 | Hs.515662 | 84878 |
| ART1 | chr11 | 3622834 | 2.29 | Hs.382188 | 417 |
| PC | chr11 | 66482492 | 2.29 | Hs.89890 | 5091 |
| LRCH1 | chr13 | 46025441 | 2.29 | Hs.656722 | 23143 |
| ART5 | chr11 | 3622834 | 2.29 | Hs.125680 | 116969 |
| ACTN1 | chr14 | 68515949 | 2.28 | Hs.509765 | 87 |
| DHRS7B | chr17 | 20887383 | 2.28 | Hs.386989 | 25979 |
| CBX8 | chr17 | 75385481 | 2.28 | Hs.387258 | 57332 |
| ATF3 | chr1 | 209170688 | 2.26 | Hs.460 | 467 |
| DUSP1 | chr5 | 172131082 | 2.26 | Hs.171695 | 1843 |
| SOCS5 | chr2 | 46837915 | 2.26 | Hs.468426 | 9655 |
| POLE3 | chr9 | 113252298 | 2.26 | Hs.108112 | 54107 |
| KCTD15 | chr19 | 38979917 | 2.26 | Hs.221873 | 79047 |
| C9orf43 | chr9 | 113252298 | 2.26 | Hs.632691 | 257169 |
| PRCC | chr1 | 153550101 | 2.25 | Hs.516948 | 5546 |
| DYNLT1 | chr6 | 159036597 | 2.25 | Hs.445999 | 6993 |
| ANKRD25 | chr19 | 11169286 | 2.25 | Hs.284208 | 25959 |
| CSDC2 | chr22 | 40281745 | 2.25 | Hs.310893 | 27254 |
| RHPN2 | chr19 | 38247999 | 2.25 | Hs.466435 | 85415 |
| SYTL3 | chr6 | 159036597 | 2.25 | Hs.436977 | 94120 |
| LDHA | chr11 | 18372510 | 2.24 | Hs.2795 | 3939 |
| R3HDML | chr20 | 42399055 | 2.24 | Hs.580807 | 140902 |
| RTP3 | chr3 | 46514407 | 2.23 | Hs.196584 | 83597 |
| NANP | chr20 | 25552742 | 2.23 | Hs.666255 | 140838 |
| DNAJB2 | chr2 | 219969266 | 2.21 | Hs.77768 | 3300 |
| RGS13 | chr1 | 189337200 | 2.21 | Hs.497220 | 6003 |
| FILIP1 | chr6 | 76259894 | 2.21 | Hs.696158 | 27145 |
| RBM25 | chr14 | 72594865 | 2.21 | Hs.531106 | 58517 |
| BEST3 | chr12 | 68369365 | 2.21 | Hs.280782 | 144453 |
| PLK3 | chr1 | 44934896 | 2.2 | Hs.632415 | 1263 |
| UVRAG | chr11 | 75203832 | 2.2 | Hs.202470 | 7405 |
| FADS2 | chr11 | 61352126 | 2.2 | Hs.502745 | 9415 |
| NUTF2 | chr16 | 66438097 | 2.2 | Hs.696342 | 10204 |
| GTF3C2 | chr2 | 27491357 | 2.19 | Hs.75782 | 2976 |
| METTL2B | chr7 | 127710525 | 2.19 | Hs.381204 | 339175 |
| ZBTB25 | chr14 | 64041074 | 2.18 | Hs.654571 | 7597 |
| ZBTB1 | chr14 | 64041074 | 2.18 | Hs.655536 | 22890 |
| DNAJB11 | chr3 | 187770491 | 2.18 | Hs.317192 | 51726 |
| TBCCD1 | chr3 | 187770491 | 2.18 | Hs.518469 | 55171 |
| JMJD1A | chr2 | 86579986 | 2.18 | Hs.557425 | 55818 |
| CD63 | chr12 | 54409365 | 2.17 | Hs.445570 | 967 |
| TSKU | chr11 | 76172332 | 2.17 | Hs.8361 | 25987 |
| CYHR1 | chr8 | 145661786 | 2.17 | Hs.459379 | 50626 |
| KIFC2 | chr8 | 145661786 | 2.17 | Hs.528713 | 90990 |
| LENG8 | chr19 | 59652204 | 2.17 | Hs.502378 | 114823 |
| GPR92 | chr12 | 6615693 | 2.16 | Hs.155538 | 57121 |
| HTR1F | chr3 | 88122510 | 2.15 | Hs.248136 | 3355 |
| PHEX | chrX | 21809999 | 2.15 | Hs.495834 | 5251 |
| ODZ1 | chrX | 123823736 | 2.15 | Hs.23796 | 10178 |
| KLF13 | chr15 | 29406012 | 2.15 | Hs.525752 | 51621 |
| LTBP3 | chr11 | 65083102 | 2.14 | Hs.289019 | 4054 |
| P2RY2 | chr11 | 72606717 | 2.14 | Hs.339 | 5029 |
| RDH13 | chr19 | 60272725 | 2.14 | Hs.327631 | 112724 |
| CCND1 | chr11 | 69164963 | 2.13 | Hs.523852 | 595 |
| NQO1 | chr16 | 68318404 | 2.12 | Hs.406515 | 1728 |
| LIF | chr22 | 28967724 | 2.12 | Hs.2250 | 3976 |
| NOTCH2 | chr1 | 120324315 | 2.12 | Hs.487360 | 4853 |
| MEP1A | chr6 | 46869161 | 2.11 | Hs.179704 | 4224 |
| RSF1 | chr11 | 77209647 | 2.11 | Hs.420229 | 51773 |
| FLJ45256 | chr16 | 24589810 | 2.11 | Hs.592028 | 400511 |
| GSS | chr20 | 33007041 | 2.1 | Hs.82327 | 2937 |
| NME1 | chr17 | 46585961 | 2.09 | Hs.463456 | 4830 |
| NPY1R | chr4 | 164612067 | 2.09 | Hs.519057 | 4886 |
| ZNF384 | chr12 | 6668812 | 2.09 | Hs.103315 | 171017 |
| ALDH3A2 | chr17 | 19492583 | 2.08 | Hs.499886 | 224 |
| CER1 | chr9 | 14713309 | 2.08 | Hs.248204 | 9350 |
| NUBP2 | chr16 | 1772668 | 2.08 | Hs.256549 | 10101 |
| SPSB3 | chr16 | 1772668 | 2.08 | Hs.592080 | 90864 |
| SSX6 | chrX | 47722515 | 2.08 | Hs.511998 | 280657 |
| CXADR | chr21 | 17807117 | 2.07 | Hs.705503 | 1525 |
| EDG6 | chr19 | 3128163 | 2.07 | Hs.662006 | 8698 |
| SPECC1 | chr17 | 20000091 | 2.07 | Hs.431045 | 92521 |
| TIPARP | chr3 | 157874938 | 2.06 | Hs.12813 | 25976 |
| CCDC123 | chr19 | 38155004 | 2.06 | Hs.599703 | 84902 |
| ITGB6 | chr2 | 160882549 | 2.05 | Hs.470399 | 3694 |
| RNPS1 | chr16 | 2258398 | 2.05 | Hs.355643 | 10921 |
| USP32 | chr17 | 55824537 | 2.05 | Hs.132868 | 84669 |
| LOC338799 | chr12 | 120700315 | 2.05 | Hs.654994 | 338799 |
| SEPT7 | chr7 | 35613713 | 2.04 | Hs.191346 | 989 |
| ITGAV | chr2 | 187280299 | 2.04 | Hs.436873 | 3685 |
| PYGL | chr14 | 50481191 | 2.03 | Hs.282417 | 5836 |
| SSR3 | chr3 | 157756283 | 2.03 | Hs.518346 | 6747 |
| DYNLL1 | chr12 | 119396624 | 2.03 | Hs.5120 | 8655 |
| SPINT2 | chr19 | 43446698 | 2.03 | Hs.31439 | 10653 |
| C19orf33 | chr19 | 43486696 | 2.03 | Hs.631544 | 64073 |
| PPP1R14A | chr19 | 43446698 | 2.03 | Hs.631569 | 94274 |
| AVPI1 | chr10 | 99437380 | 2.02 | Hs.23918 | 60370 |
| TNMD | chrX | 99646141 | 2.02 | Hs.132957 | 64102 |
| ADM2 | chr22 | 49213275 | 2.02 | Hs.449099 | 79924 |
| SHOC2 | chr10 | 112669380 | 2.01 | Hs.104315 | 8036 |
| BRWD1 | chr21 | 39607733 | 2.01 | Hs.654740 | 54014 |
| BICD1 | chr12 | 32151072 | 2 | Hs.505202 | 636 |
| DBT | chr1 | 100411871 | 2 | Hs.633217 | 1629 |
| DLX4 | chr17 | 45401200 | 2 | Hs.591167 | 1748 |
| KLF10 | chr8 | 103737230 | 2 | Hs.435001 | 7071 |
| ETHE1 | chr19 | 48728748 | 2 | Hs.7486 | 23474 |
| RNF19A | chr8 | 101391492 | 2 | Hs.292882 | 25897 |
| GEMIN8 | chrX | 13808398 | 2 | Hs.592237 | 54960 |
| B4GALNT2 | chr17 | 44565309 | 2 | Hs.374679 | 124872 |
| ZNF575 | chr19 | 48728748 | 2 | Hs.213534 | 284346 |
| NOTCH2NL | chr1 | 142698118 | 2 | Hs.655156 | 388677 |
| PNKD | chr2 | 218983493 | 1.99 | Hs.163924 | 4306 |
| SLC16A1 | chr1 | 113210809 | 1.99 | Hs.75231 | 6566 |
| JPH2 | chr20 | 42250124 | 1.99 | Hs.441737 | 57158 |
| CDK7 | chr5 | 68565869 | 1.98 | Hs.184298 | 1022 |
| RARA | chr17 | 35751844 | 1.98 | Hs.654583 | 5914 |
| GNL2 | chr1 | 37730969 | 1.98 | Hs.75528 | 29889 |
| MLL5 | chr7 | 104248163 | 1.98 | Hs.592262 | 55904 |
| DAND5 | chr19 | 12941151 | 1.98 | Hs.331981 | 199699 |
| DAB2 | chr5 | 39461275 | 1.97 | Hs.481980 | 1601 |
| PHKA2 | chrX | 18762736 | 1.97 | Hs.54941 | 5256 |
| PNMA1 | chr14 | 73250709 | 1.97 | Hs.194709 | 9240 |
| LDOC1 | chrX | 139997633 | 1.97 | Hs.45231 | 23641 |
| BRF2 | chr8 | 37826824 | 1.97 | Hs.705411 | 55290 |
| KLC1 | chr14 | 103164959 | 1.96 | Hs.20107 | 3831 |
| RPH3AL | chr17 | 202819 | 1.96 | Hs.651925 | 9501 |
| MTP18 | chr22 | 29146372 | 1.96 | Hs.656909 | 51537 |
| MPP5 | chr14 | 66777937 | 1.96 | Hs.652312 | 64398 |
| IL6R | chr1 | 151190756 | 1.95 | Hs.695954 | 3570 |
| PFTK1 | chr7 | 89983182 | 1.95 | Hs.430742 | 5218 |
| HLTF | chr3 | 150287144 | 1.95 | Hs.3068 | 6596 |
| MAP3K7IP2 | chr6 | 149680899 | 1.95 | Hs.269775 | 23118 |
| TPTE2 | chr13 | 18976222 | 1.95 | Hs.377488 | 93492 |
| FAM33A | chr17 | 54587430 | 1.95 | Hs.463607 | 348235 |
| HIBCH | chr2 | 191010193 | 1.94 | Hs.656685 | 26275 |
| AXL | chr19 | 46416604 | 1.93 | Hs.590970 | 558 |
| ARL4C | chr2 | 235188089 | 1.93 | Hs.699342 | 10123 |
| KIAA1161 | chr9 | 34366716 | 1.93 | Hs.522083 | 57462 |
| SLC25A28 | chr10 | 101371020 | 1.93 | Hs.403790 | 81894 |
| KIAA1737 | chr14 | 76634055 | 1.93 | Hs.22452 | 85457 |
| CTPS | chr1 | 41114265 | 1.92 | Hs.473087 | 1503 |
| DBP | chr19 | 53832851 | 1.92 | Hs.414480 | 1628 |
| C20orf29 | chr20 | 3749546 | 1.92 | Hs.104806 | 55317 |
| KIAA1609 | chr16 | 83096119 | 1.92 | Hs.288274 | 57707 |
| EPB41L1 | chr20 | 34137514 | 1.91 | Hs.437422 | 2036 |
| LBR | chr1 | 221923034 | 1.91 | Hs.435166 | 3930 |
| MDFI | chr6 | 41714646 | 1.91 | Hs.520119 | 4188 |
| SPIN1 | chr9 | 88232992 | 1.91 | Hs.146804 | 10927 |
| TRIM31 | chr6 | 30189282 | 1.91 | Hs.493275 | 11074 |
| DDT | chr22 | 22646286 | 1.9 | Hs.656723 | 1652 |
| GSTT2 | chr22 | 22646286 | 1.9 | Hs.654462 | 2953 |
| JARID1A | chr12 | 368774 | 1.9 | Hs.654806 | 5927 |
| PLA2G7 | chr6 | 46811879 | 1.9 | Hs.584823 | 7941 |
| SLC7A5 | chr16 | 86460921 | 1.9 | Hs.513797 | 8140 |
| PGS1 | chr17 | 73886212 | 1.9 | Hs.654671 | 9489 |
| ARHGAP11A | chr15 | 30694588 | 1.9 | Hs.591130 | 9824 |
| SH3TC1 | chr4 | 8319339 | 1.9 | Hs.479116 | 54436 |
| MKI67IP | chr2 | 122210987 | 1.9 | Hs.367842 | 84365 |
| JUB | chr14 | 22521653 | 1.89 | Hs.655832 | 84962 |
| ARHGAP5 | chr14 | 31616221 | 1.88 | Hs.592313 | 394 |
| CHRNB2 | chr1 | 151352809 | 1.88 | Hs.2306 | 1141 |
| HNRPK | chr9 | 83824736 | 1.88 | Hs.695973 | 3190 |
| CCDC9 | chr19 | 52451786 | 1.88 | Hs.227782 | 26093 |
| TMEM132A | chr11 | 60448712 | 1.88 | Hs.118552 | 54972 |
| UBE2Q1 | chr1 | 151352809 | 1.88 | Hs.607928 | 55585 |
| MUS81 | chr11 | 65384681 | 1.88 | Hs.288798 | 80198 |
| MGC34761 | chr16 | 68765484 | 1.88 | Hs.556045 | 283971 |
| SH3BGR | chr21 | 39745056 | 1.87 | Hs.473847 | 6450 |
| KIAA0174 | chr16 | 70486921 | 1.87 | Hs.232194 | 9798 |
| DSTN | chr20 | 17498671 | 1.87 | Hs.304192 | 11034 |
| THAP8 | chr19 | 41237332 | 1.87 | Hs.350209 | 199745 |
| WDR62 | chr19 | 41237332 | 1.87 | Hs.116244 | 284403 |
| PNRC1 | chr6 | 89843050 | 1.86 | Hs.75969 | 10957 |
| NHS | chrX | 17152896 | 1.85 | Hs.201623 | 4810 |
| TXN | chr9 | 110098798 | 1.85 | Hs.435136 | 7295 |
| CATSPER2 | chr15 | 41773314 | 1.85 | Hs.662284 | 117155 |
| HSPA2 | chr14 | 64071672 | 1.84 | Hs.432648 | 3306 |
| PHLDA2 | chr11 | 2907764 | 1.84 | Hs.154036 | 7262 |
| KIAA1576 | chr16 | 76379759 | 1.84 | Hs.461405 | 57687 |
| KLC4 | chr6 | 43135578 | 1.84 | Hs.655123 | 89953 |
| ADPRHL1 | chr13 | 113151730 | 1.84 | Hs.98669 | 113622 |
| HTRA4 | chr8 | 38950832 | 1.84 | Hs.661014 | 203100 |
| SLC1A4 | chr2 | 65128390 | 1.83 | Hs.654352 | 6509 |
| RABGAP1 | chr9 | 122876454 | 1.83 | Hs.271341 | 23637 |
| PGM2L1 | chr11 | 73787093 | 1.83 | Hs.26612 | 283209 |
| OR8H3 | chr11 | 55646082 | 1.83 | Hs.553745 | 390152 |
| NR6A1 | chr9 | 124618664 | 1.82 | Hs.586460 | 2649 |
| TPSG1 | chr16 | 1369402 | 1.81 | Hs.592076 | 25823 |
| ZNF34 | chr8 | 145983344 | 1.81 | Hs.631854 | 80778 |
| C19orf36 | chr19 | 2047107 | 1.81 | Hs.424049 | 113177 |
| MOBKL2A | chr19 | 2047107 | 1.81 | Hs.86912 | 126308 |
| RAB3IL1 | chr11 | 61442028 | 1.8 | Hs.13759 | 5866 |
| DMTF1 | chr7 | 86426447 | 1.8 | Hs.654981 | 9988 |
| ACAA2 | chr18 | 45594297 | 1.8 | Hs.200136 | 10449 |
| ANKRD10 | chr13 | 110365683 | 1.8 | Hs.525163 | 55608 |
| MTUS1 | chr8 | 17702930 | 1.8 | Hs.7946 | 57509 |
| C14orf80 | chr14 | 105027257 | 1.8 | Hs.72363 | 283643 |
| MAP3K8 | chr10 | 30763163 | 1.79 | Hs.432453 | 1326 |
| MEF2A | chr15 | 97923746 | 1.79 | Hs.268675 | 4205 |
| PDE3A | chr12 | 20413110 | 1.79 | Hs.591150 | 5139 |
| SIRT1 | chr10 | 69314191 | 1.79 | Hs.369779 | 23411 |
| WDR34 | chr9 | 128498258 | 1.79 | Hs.495240 | 89891 |
| SELM | chr22 | 29827952 | 1.79 | Hs.55940 | 140606 |
| VWA2 | chr10 | 115988802 | 1.79 | Hs.197741 | 340706 |
| LOC388272 | chr16 | 45422509 | 1.79 | Hs.705603 | 388272 |
| ACACA | chr17 | 32790344 | 1.78 | Hs.160556 | 31 |
| PRKAR1A | chr17 | 64020027 | 1.78 | Hs.280342 | 5573 |
| PPP1R11 | chr6 | 30142911 | 1.78 | Hs.82887 | 6992 |
| C2orf25 | chr2 | 150270233 | 1.78 | Hs.5324 | 27249 |
| SLC25A23 | chr19 | 6411090 | 1.78 | Hs.356231 | 79085 |
| CRB3 | chr19 | 6411090 | 1.78 | Hs.150319 | 92359 |
| GPR39 | chr2 | 133007999 | 1.77 | Hs.432395 | 2863 |
| PPM1B | chr2 | 44307419 | 1.77 | Hs.416769 | 5495 |
| TXK | chr4 | 47977961 | 1.77 | Hs.479669 | 7294 |
| CIB1 | chr15 | 88578247 | 1.77 | Hs.135471 | 10519 |
| OR6C2 | chr12 | 54132054 | 1.77 | Hs.524483 | 341416 |
| DLX3 | chr17 | 45427621 | 1.76 | Hs.134194 | 1747 |
| EWSR1 | chr22 | 27988509 | 1.76 | Hs.374477 | 2130 |
| H3F3B | chr17 | 71292676 | 1.76 | Hs.180877 | 3021 |
| PFKFB3 | chr10 | 6284991 | 1.76 | Hs.195471 | 5209 |
| STYX | chr14 | 52266644 | 1.76 | Hs.364980 | 6815 |
| TRDN | chr6 | 123999620 | 1.76 | Hs.654601 | 10345 |
| KIAA0182 | chr16 | 84202681 | 1.76 | Hs.461647 | 23199 |
| RHBDD3 | chr22 | 27988509 | 1.76 | Hs.106730 | 25807 |
| PLEKHG3 | chr14 | 64240604 | 1.76 | Hs.509637 | 26030 |
| AXUD1 | chr3 | 39170280 | 1.76 | Hs.370950 | 64651 |
| GSTP1 | chr11 | 67107691 | 1.75 | Hs.523836 | 2950 |
| MAP2K1 | chr15 | 64466061 | 1.75 | Hs.145442 | 5604 |
| CTDSP2 | chr12 | 56527165 | 1.75 | Hs.524530 | 10106 |
| DDX39 | chr19 | 14391221 | 1.75 | Hs.311609 | 10212 |
| RAB40C | chr16 | 579936 | 1.75 | Hs.459630 | 57799 |
| INTS4 | chr11 | 77383253 | 1.75 | Hs.533723 | 92105 |
| PDHX | chr11 | 34894499 | 1.74 | Hs.502315 | 8050 |
| APIP | chr11 | 34894499 | 1.74 | Hs.447794 | 51074 |
| LENG9 | chr19 | 59666447 | 1.74 | Hs.590976 | 94059 |
| KCNN4 | chr19 | 48976906 | 1.73 | Hs.10082 | 3783 |
| RFX1 | chr19 | 13977968 | 1.73 | Hs.655215 | 5989 |
| TNIP1 | chr5 | 150441333 | 1.73 | Hs.543850 | 10318 |
| ANK2 | chr4 | 114328246 | 1.72 | Hs.620557 | 287 |
| DIAPH2 | chrX | 95944884 | 1.72 | Hs.696382 | 1730 |
| TSC22D3 | chrX | 106766377 | 1.72 | Hs.522074 | 1831 |
| ZNF155 | chr19 | 49175336 | 1.72 | Hs.502127 | 7711 |
| PAK6 | chr15 | 38318883 | 1.72 | Hs.513645 | 56924 |
| RFT1 | chr3 | 53139542 | 1.72 | Hs.631910 | 91869 |
| ME1 | chr6 | 84197765 | 1.71 | Hs.21160 | 4199 |
| PSMD13 | chr11 | 226555 | 1.71 | Hs.134688 | 5719 |
| VAPB | chr20 | 56397251 | 1.71 | Hs.182625 | 9217 |
| COTL1 | chr16 | 83210136 | 1.71 | Hs.289092 | 23406 |
| SIRT3 | chr11 | 226555 | 1.71 | Hs.592292 | 23410 |
| TRPM7 | chr15 | 48766004 | 1.71 | Hs.512894 | 54822 |
| PPP2R2D | chr10 | 133597623 | 1.71 | Hs.380372 | 55844 |
| CARD14 | chr17 | 75776027 | 1.71 | Hs.675480 | 79092 |
| HAVCR2 | chr5 | 156469039 | 1.71 | Hs.616365 | 84868 |
| REEP3 | chr10 | 64895339 | 1.71 | Hs.499833 | 221035 |
| GPLD1 | chr6 | 24597758 | 1.7 | Hs.591810 | 2822 |
| MAP3K3 | chr17 | 59053262 | 1.7 | Hs.29282 | 4215 |
| OR5I1 | chr11 | 55460238 | 1.7 | Hs.533706 | 10798 |
| PLEK2 | chr14 | 66948624 | 1.7 | Hs.170473 | 26499 |
| EEF2K | chr16 | 22124958 | 1.7 | Hs.498892 | 29904 |
| COX18 | chr4 | 74301004 | 1.7 | Hs.356697 | 285521 |
| ELA2 | chr19 | 802088 | 1.69 | Hs.99863 | 1991 |
| SFTPD | chr10 | 81699125 | 1.69 | Hs.253495 | 6441 |
| DGCR6 | chr22 | 17268521 | 1.69 | Hs.474185 | 8214 |
| DENR | chr12 | 121762049 | 1.69 | Hs.22393 | 8562 |
| WDR53 | chr3 | 197783676 | 1.69 | Hs.385865 | 348793 |
| FSHB | chr11 | 30209672 | 1.68 | Hs.36975 | 2488 |
| MMP12 | chr11 | 102251135 | 1.68 | Hs.1695 | 4321 |
| NRL | chr14 | 23632852 | 1.68 | Hs.652297 | 4901 |
| PON2 | chr7 | 94709117 | 1.68 | Hs.530077 | 5445 |
| RBMS2 | chr12 | 55201788 | 1.68 | Hs.645521 | 5939 |
| WDR19 | chr4 | 39005858 | 1.68 | Hs.438482 | 57728 |
| MET | chr7 | 115906181 | 1.67 | Hs.132966 | 4233 |
| VDAC1 | chr5 | 133368997 | 1.67 | Hs.519320 | 7416 |
| PPP2R3C | chr14 | 34661204 | 1.67 | Hs.530712 | 55012 |
| H2AFV | chr7 | 44660767 | 1.67 | Hs.488189 | 94239 |
| CTGF | chr6 | 132314041 | 1.66 | Hs.591346 | 1490 |
| GIPR | chr19 | 50863038 | 1.66 | Hs.658534 | 2696 |
| MCM6 | chr2 | 136467864 | 1.66 | Hs.444118 | 4175 |
| FZD1 | chr7 | 90538456 | 1.66 | Hs.94234 | 8321 |
| MAN1B1 | chr9 | 137256976 | 1.66 | Hs.591887 | 11253 |
| MAF1 | chr8 | 145230795 | 1.66 | Hs.19673 | 84232 |
| FCHO2 | chr5 | 72287592 | 1.66 | Hs.165762 | 115548 |
| ANXA5 | chr4 | 122975564 | 1.65 | Hs.480653 | 308 |
| SF3B2 | chr11 | 65578885 | 1.65 | Hs.406423 | 10992 |
| ATF7 | chr12 | 52306432 | 1.65 | Hs.12286 | 11016 |
| OSBPL1A | chr18 | 20231819 | 1.65 | Hs.370725 | 114876 |
| CYP2C19 | chr10 | 96512336 | 1.64 | Hs.282409 | 1557 |
| MAP3K4 | chr6 | 161383090 | 1.64 | Hs.390428 | 4216 |
| MYOG | chr1 | 199786477 | 1.64 | Hs.2830 | 4656 |
| PCBP2 | chr12 | 52132089 | 1.64 | Hs.546271 | 5094 |
| PIN4 | chrX | 71184411 | 1.64 | Hs.655623 | 5303 |
| WNT9A | chr1 | 224442716 | 1.64 | Hs.149504 | 7483 |
| OR10W1 | chr11 | 57792015 | 1.64 | Hs.531507 | 81341 |
| GAS2L3 | chr12 | 99469912 | 1.64 | Hs.20575 | 283431 |
| C5orf13 | chr5 | 111121894 | 1.63 | Hs.36053 | 9315 |
| ARL4A | chr7 | 12499546 | 1.63 | Hs.245540 | 10124 |
| MESDC1 | chr15 | 79080332 | 1.63 | Hs.513071 | 59274 |
| P4HA3 | chr11 | 73700313 | 1.63 | Hs.660541 | 283208 |
| PTGS2 | chr1 | 183381315 | 1.62 | Hs.196384 | 5743 |
| TEC | chr4 | 48184252 | 1.62 | Hs.479670 | 7006 |
| SLCO1B3 | chr12 | 20860118 | 1.62 | Hs.504966 | 28234 |
| VCPIP1 | chr8 | 67742095 | 1.62 | Hs.632066 | 80124 |
| FAH | chr15 | 78232298 | 1.61 | Hs.73875 | 2184 |
| PICALM | chr11 | 85458016 | 1.61 | Hs.163893 | 8301 |
| CIT | chr12 | 118778100 | 1.61 | Hs.119594 | 11113 |
| ANKRD2 | chr15 | 50648571 | 1.61 | Hs.73708 | 26287 |
| LUC7L2 | chr7 | 138501589 | 1.61 | Hs.370475 | 51631 |
| C21orf29 | chr21 | 44941617 | 1.61 | Hs.660703 | 54084 |
| FBXO17 | chr19 | 44158587 | 1.61 | Hs.531770 | 115290 |
| NEBL | chr10 | 21503204 | 1.6 | Hs.5025 | 10529 |
| FASTKD2 | chr2 | 207455612 | 1.6 | Hs.84429 | 22868 |
| TPCN1 | chr12 | 112122259 | 1.6 | Hs.524763 | 53373 |
| MDH1B | chr2 | 207455612 | 1.6 | Hs.147816 | 130752 |
| GATA6 | chr18 | 18003213 | 1.59 | Hs.514746 | 2627 |
| CCPG1 | chr15 | 53487891 | 1.59 | Hs.612814 | 9236 |
| RBM7 | chr11 | 113776716 | 1.59 | Hs.533736 | 10179 |
| PPIL2 | chr22 | 20345038 | 1.59 | Hs.438587 | 23759 |
| MTERFD3 | chr12 | 105883808 | 1.59 | Hs.5009 | 80298 |
| RTN3 | chr11 | 63205567 | 1.58 | Hs.473761 | 10313 |
| DKFZP564J0863 | chr11 | 63205567 | 1.58 | Hs.356719 | 25923 |
| CTDSPL2 | chr15 | 42507048 | 1.58 | Hs.646495 | 51496 |
| GALNT10 | chr5 | 153549955 | 1.58 | Hs.651323 | 55568 |
| ADCK4 | chr19 | 45914422 | 1.58 | Hs.130712 | 79934 |
| ITPKC | chr19 | 45914422 | 1.58 | Hs.515415 | 80271 |
| OR4K17 | chr14 | 19654807 | 1.58 | Hs.553765 | 390436 |
| PIM3 | chr22 | 48674681 | 1.58 | Hs.530381 | 415116 |
| GALNAC4S | chr10 | 125843159 | 1.58 | Hs.287537 | 51363 |
| BCL3 | chr19 | 49943891 | 1.57 | Hs.31210 | 602 |
| CALR | chr19 | 12910314 | 1.57 | Hs.515162 | 811 |
| FARSA | chr19 | 12910314 | 1.57 | Hs.23111 | 2193 |
| GCLM | chr1 | 94087220 | 1.57 | Hs.315562 | 2730 |
| KPNA4 | chr3 | 161766353 | 1.57 | Hs.288193 | 3840 |
| PSMA1 | chr11 | 14498596 | 1.57 | Hs.102798 | 5682 |
| HIP1R | chr12 | 121844141 | 1.57 | Hs.524815 | 9026 |
| GALNT7 | chr4 | 174464390 | 1.57 | Hs.548088 | 51809 |
| DAP3 | chr1 | 152471524 | 1.56 | Hs.516746 | 7818 |
| RGS20 | chr8 | 54956162 | 1.56 | Hs.368733 | 8601 |
| CAMTA1 | chr1 | 7068594 | 1.56 | Hs.397705 | 23261 |
| MYEF2 | chr15 | 46257669 | 1.56 | Hs.6638 | 50804 |
| YY1AP1 | chr1 | 152471524 | 1.56 | Hs.584927 | 55249 |
| CENPN | chr16 | 79598402 | 1.56 | Hs.55028 | 55839 |
| ZNF223 | chr19 | 49247871 | 1.55 | Hs.279840 | 7766 |
| CCNK | chr14 | 99017359 | 1.55 | Hs.705475 | 8812 |
| NPC2 | chr14 | 74029931 | 1.55 | Hs.433222 | 10577 |
| SNX5 | chr20 | 17897835 | 1.55 | Hs.316890 | 27131 |
| SETD3 | chr14 | 99017359 | 1.55 | Hs.510407 | 84193 |
| ISCA2 | chr14 | 74029931 | 1.55 | Hs.702169 | 122961 |
| ADH1B | chr4 | 100600256 | 1.54 | Hs.4 | 125 |
| PTK2B | chr8 | 27239042 | 1.54 | Hs.491322 | 2185 |
| PAFAH1B2 | chr11 | 116520415 | 1.54 | Hs.696131 | 5049 |
| PRCP | chr11 | 82289389 | 1.54 | Hs.523936 | 5547 |
| SEPP1 | chr5 | 42847728 | 1.54 | Hs.275775 | 6414 |
| SLC12A4 | chr16 | 66559989 | 1.54 | Hs.10094 | 6560 |
| IQCE | chr7 | 2368674 | 1.54 | Hs.520627 | 23288 |
| RTDR1 | chr22 | 21736652 | 1.54 | Hs.526920 | 27156 |
| C12orf5 | chr12 | 4300744 | 1.54 | Hs.504545 | 57103 |
| TFB2M | chr1 | 243055944 | 1.54 | Hs.7395 | 64216 |
| C7orf27 | chr7 | 2368674 | 1.54 | Hs.520623 | 221927 |
| CTNNA1 | chr5 | 138116850 | 1.53 | Hs.534797 | 1495 |
| EIF6 | chr20 | 33335954 | 1.53 | Hs.654848 | 3692 |
| POLG | chr15 | 87679341 | 1.53 | Hs.702153 | 5428 |
| CSAD | chr12 | 51860758 | 1.53 | Hs.279815 | 51380 |
| SMPD4 | chr2 | 130655729 | 1.53 | Hs.516450 | 55627 |
| POLR3B | chr12 | 105254045 | 1.53 | Hs.62696 | 55703 |
| MZT2B | chr2 | 130655729 | 1.53 | Hs.469925 | 80097 |
| ZNF740 | chr12 | 51860758 | 1.53 | Hs.524458 | 283337 |
| RAB11FIP3 | chr16 | 415058 | 1.52 | Hs.531642 | 9727 |
| SMURF2 | chr17 | 60088998 | 1.52 | Hs.705442 | 64750 |
| DHRS4L2 | chr14 | 23528135 | 1.52 | Hs.647569 | 317749 |
| CTCF | chr16 | 66154206 | 1.51 | Hs.368367 | 10664 |
| MRPL39 | chr21 | 25901704 | 1.51 | Hs.420696 | 54148 |
| PARP16 | chr15 | 63366204 | 1.51 | Hs.30634 | 54956 |
| ARPC5L | chr9 | 124711259 | 1.51 | Hs.132499 | 81873 |
| RELA | chr11 | 65187296 | 1.5 | Hs.502875 | 5970 |
| DHX38 | chr16 | 70685291 | 1.5 | Hs.570079 | 9785 |
| POLR1D | chr13 | 27092997 | 1.5 | Hs.507584 | 51082 |
| PEX26 | chr22 | 16934732 | 1.5 | Hs.517400 | 55670 |
| LNX2 | chr13 | 27092997 | 1.5 | Hs.132359 | 222484 |
