# Supplementary material for: High Throughput Determination of TGFβ1/SMAD3 Targets in A549 Lung Epithelial Cells
Source: PLoS One. 2011 May 20;6(5):e20319. doi: 10.1371/journal.pone.0020319 (PMC3098871; doi:10.1371/journal.pone.0020319)
Supplement: Table S3 — SMAD3 Target Genes that are Changed in IPF Lungs. (DOCX) [file pone.0020319.s004.docx]

**Table S3. SMAD3 Target Genes that are Changed in IPF Lungs**

| **Gene ID** | **UniGene ID** | **Entrez ID** | **Chromosome** | **Peak Height^1^** | ***q* Value IPF/CTRL^2^** | **Fold Change IPF/CTRL^3^** |
| --- | --- | --- | --- | --- | --- | --- |
| COL7A1 | Hs.476218 | 1294 | chr3 | 4.63 | 0.000 | 2.9 |
| SMAD7 | Hs.465087 | 4092 | chr18 | 3.91 | 2.234 | 0.6 |
| S100A2 | Hs.516484 | 6273 | chr1 | 3.31 | 0.000 | 8.1 |
| RRAS | Hs.515536 | 6237 | chr19 | 3.30 | 0.466 | 0.6 |
| MYO1D | Hs.658000 | 4642 | chr17 | 3.23 | 0.000 | 1.3 |
| TRIB1 | Hs.444947 | 10221 | chr8 | 3.17 | 2.772 | 0.6 |
| QPCT | Hs.79033 | 25797 | chr2 | 2.67 | 4.272 | 1.3 |
| C14orf79 | Hs.27183 | 122616 | chr14 | 2.66 | 3.382 | 1.5 |
| TAGLN | Hs.632099 | 6876 | chr11 | 2.62 | 0.316 | 1.5 |
| C14orf43 | Hs.656506 | 91748 | chr14 | 2.60 | 0.000 | 0.6 |
| SH2D4A | Hs.303208 | 63898 | chr8 | 2.54 | 0.466 | 0.7 |
| PDE7B | Hs.652367 | 27115 | chr6 | 2.51 | 1.766 | 1.4 |
| ITGB1 | Hs.695946 | 3688 | chr10 | 2.50 | 1.766 | 0.4 |

1. The peak heights of SMAD3 binding peaks for each of the target genes of the ChIP-on-chip analysis.
2. The *q* value of gene expression microarray analysis of IPF and control lungs. A *q* value of 5, which corresponds to a 5% false discovery rate, was used as a cutoff of statistical significance in microarray data.
3. The fold change of differentially expressed genes between IPF and control lungs.
